# High-parameter immunophenotyping reveals distinct immune cell profiles in pruritic dogs and cats

**DOI:** 10.3389/fvets.2024.1498964

**Published:** 2025-01-22

**Authors:** Erin McDonald, Eric Kehoe, Darcy Deines, Mary McCarthy, Brie Wright, Susan Huse

**Affiliations:** Veterinary Medicine Research and Development (VMRD), Zoetis Inc, Fort Collins, CO, United States

**Keywords:** flow cytometry, pruritis, immunophenotyping, machine learning, artificial intelligence, unsupervised clustering, canine, feline

## Abstract

**Introduction:**

Immunophenotyping is a powerful tool for grading disease severity, aiding in diagnosis, predicting clinical response, and guiding the development of novel therapeutics.

**Methods:**

This pilot study employs high parameter immunophenotyping panels (15 markers for dog, 12 for cat) and leverages unsupervised clustering to identify immune cell populations. Our analysis uses machine learning and statistical algorithms to perform unsupervised clustering, multiple visualizations, and statistical analysis of high parameter flow cytometry data. This method reduces user bias and precisely identifies cell populations, demonstrating its potential to detect variations and differentiate populations effectively. To enhance our understanding of cat and dog biology and test the unsupervised clustering approach on real-world samples, we performed in-depth profiling of immune cell populations in blood collected from client-owned and laboratory animals [dogs (*n* = 55) and cats (*n* = 68)]. These animals were categorized based on pruritic behavior or routine check-ups (non-pruritic controls).

**Results:**

Unsupervised clustering revealed various immune cell populations, including T-cell subsets distinguished by CD62L expression and distinct monocyte subsets. Notably, there were significant differences in monocyte subsets between pruritic and non-pruritic animals. Pruritic dogs and cats showed significant shifts in CD62LHi T-cell subsets compared to non-pruritic controls, with opposite trends observed between pruritic cats and dogs.

**Discussion:**

These findings underscore the importance of advancing veterinary immunophenotyping, expanding our knowledge about marker expression on circulating immune cells and driving progress in understanding veterinary-specific biology and uncovering new insights into various conditions and diseases.

## Introduction

Flow cytometry is a technique for identifying, characterizing, and quantifying cells by measuring the expression of cell-specific markers. Known as immunophenotyping, this process reveals critical insights into disease mechanisms and facilitates biomarker discovery. While extensively used in human clinical research, its application in veterinary research is growing, particularly in veterinary oncology where it is being used to characterize certain cancers (1). However, veterinary immunophenotyping panels have traditionally lagged behind their human counterparts due to a limited availability of species-specific antibodies and fewer available fluorophore options for these species-specific antibodies. To address this gap, we developed a high parameter immunophenotyping panel by identifying cross-species antibodies and utilizing spectral flow cytometry to overcome fluorophore limitations.

High parameter panels allow each cell to be characterized by multiple markers. Manual gating, however, limits analysis to two markers at a time and relies heavily on user expertise and subjective judgement, introducing bias and variability. As the number of markers increases, data complexity grows. Unsupervised clustering, powered by machine learning, reduces user bias, providing an objective analysis without pre-conceived notions on how cell populations should be gated. These methods group cells based on their similarities in marker expression across all markers at once, without relying on subjective human gating, which increases the reliability of the results. Furthermore, clustering can uncover patterns and relationships in the data that might not be apparent through manual analysis, and this can lead to the discovery of rare and/or novel cell populations.

Our real-world samples allowed us to identify differences in the immune cell landscape of the blood between animals with active pruritic behavior, a common clinical sign associated with canine and feline allergy, and those undergoing routine health checks (non-pruritic controls). In humans and mice, effector T-cells, including CD4 and CD8 T cells, are pivotal in pruritic behavior, especially in atopic dermatitis (AD). T helper 2 (Th2) type cytokines potentiate interactions between nerves and T lymphocytes. For example, interleukin-31 (IL-31), a pruritogenic cytokine produced by Th2 cells (3, 4), is significantly upregulated in pruritic AD patients, leading to severe itching. In addition, neutrophils and monocytes also play significant roles in pruritus. In an AD mouse model, neutrophils were shown to promote itch by triggering CXCR3-dependent pathway in sensory neurons, causing skin hyperinnervation, and increasing expression of itch signaling molecules. Neutrophil depletion in this model also reduces scratching (5). Monocytes, particularly intermediate monocytes, are linked to the severity of uraemic pruritus in human patients (6). Monocytes release many proinflammatory cytokines, including Oncostatin M (OSM). While OSM does not directly cause itch, it is hypothesized to induce hypersensitivity by enhancing neuronal responses to histamine and leukotriene. Many inflammatory skin diseases, including cutaneous T-cell lymphoma, psoriasis, and atopic dermatitis, show an overexpression of OSM in skin lesions (7, 8).

This study provides an updated, comprehensive list of immunophenotyping markers for cats and dogs. A limitation of this study is that the only inclusion criteria was active pruritic behavior. However, this focus is justified as pruritis is a cardinal sign of allergic and atopic dermatitis, which guided our hypotheses. Pruritic behavior in dogs can also be caused by lymphomas. We ensured BioIVT included cancer/lypmhoma diagnoses in the metadata, and none of the samples were from animals with these conditions. Future studies could benefit from well-defined case criteria to compare pruritic behavior due to known diagnoses. Identifying cell types and their functions during pruritis will lead to more complex hypotheses about the role of these immune cell subsets in canine and feline allergies. Furthermore, this study demonstrates that flow cytometric analysis can detect potentially significant alterations of leukocyte populations independently of the leukogram. This data can help detect variations in cellular subsets across different disease states, treatments, or disease models and this work underscores the importance of advancing veterinary immunophenotyping, driving excitement and progress in understanding veterinary-specific biology and uncovering new insights into disease.

## Materials and methods

### Canine and feline patients

#### Ethics

Written informed owner consent was obtained for all client-owned specimens. We worked with BioIVT, a leading global provider of high-quality biological specimens, to source animal biospecimens from recruited veterinary clinics. BioIVT recruited client owned animals (*n* = 37 dogs, *n* = 42 cats); these animals fell into one of two categories: pruritic behavior (*n* = 29 dogs, *n* = 42 cats) or non-pruritic (routine checkup; *n* = 8 dogs). Additional non-pruritic control blood (*n* = 25 cats; *n* = 10 beagles) was collected from laboratory animals maintained by BioIVT for routine use to supply biospecimens to life science and diagnostic industries. Samples from five pruritic beagles with presumptive diagnoses of canine atopic dermatitis were obtained from an internal colony whose clinical signs were managed by internal veterinarians. Additional non-pruritic blood was collected from three laboratory beagles (also housed internally). All animal sample collection was approved by the Institutional Animal Care and Use Committee (IACUC). BioIVT collected metadata (gender, age, weight, breed, formal diagnosis, current medications, and start date of pruritic symptoms) when possible, but this information was missing for most pruritic animals. Only four cats (of 42 pruritic cats) had a formal diagnosis for their pruritic behavior: two cats had fleas, and two cats had a food allergy. Of the dogs identified by BioIVT, three dogs had a formal diagnosis for their pruritic behavior: two dogs were officially diagnosed with canine Atopic Dermatitis (cAD), and one of those dogs also had a separate food allergy. The third dog had a food allergy. Figures 2A, 6A have a breakdown of the pruritic and non-pruritic animals.

### Sample handling, flow cytometry staining, and spectral data acquisition

One to three mL of whole blood was collected in K2EDTA tubes. It was shipped priority overnight at ambient temperature to Fort Collins, CO. Once the samples were received, the blood was gently mixed by inverting. A complete blood cell count (CBC), including white blood cell (WBC) differential, red blood cell (RBC) and platelet counts, platelet measurements and hemoglobin concentration was completed (Element HT5 automated hematology analyzer, Heska). Basophils were not reliably detected in all animals and were not reported. Background check and daily quality control were performed each day before running any samples. All measured parameters fell within normal range. Although all measured parameters fell within normal range and were not flagged as abnormal, there were significant differences noted between non-pruritic and pruritic animals.

Whole blood (100 μL for canine and 200 μL for feline) was added to a 96-well deep well plate. Canine blood was incubated with Zombie NIR Fixable viability stain (BioLegend) and feline blood was incubated with Live-or-Dye 510/550 Fixable viability stain (Biotium). Next, 50 μL of antibody master mix was added directly to the blood and mixed and left at 4°C (protected from light) for a total of 30 minutes; for feline blood, APC-R700 Streptavidin was added for the final 15 min of staining. After staining, 1 mL of BD FACS buffer was added to the deep well and the whole blood was pelleted at 200 rcf for 5 min. The supernatant was aspirated and then resuspended in 1 mL of 1x RT 1-step Fix/Lyse Buffer (Invitrogen, Waltham, MA, USA). After 15 min, the lysed blood was spun at 200 rcf for 5 min and the supernatant was aspirated completely. The remaining cell pellet was resuspended in 200 μL of BD FACS buffer. Samples were run within 72 h of fixing. Sample data was collected on a 5-laser Aurora spectral cytometer (Cytek). Prior to running samples, daily instrument quality control was performed. One pruritic feline sample and two pruritic canine samples were not stained for flow analysis due to timing and/or staining issues. Therefore, all flow cytometry plots represent 41 pruritic feline and 33 pruritic canine samples.

Immunophenotyping panels were designed and optimized to identify cell populations in canine and feline blood (see Supplementary Figures 2C, 4C for NxN matrices used to evaluate unmixing for canine and feline panels, respectively). Cross-reactive anti-human and anti-mouse antibodies were identified from literature and from internal testing. All cross-reactive antibodies were validated by confirming they specifically bound to their target cell populations by co-expression of other defining markers. Furthermore, each antibody was titrated. A feline anti-CD25 was not included because the clone (9F23) is not commercially available. A canine anti-TCRγδ was not included due to limited fluorophore availability. Single-color controls were run on a combination of cells and UltraComp eBeads Plus (ThermoFisher) for unmixing. Flow Cytometry Standard (FCS) files were exported and analyzed using FlowJo^TM^ Software and cleaned up (see Supplementary Figures 2A, 4A for gating strategy to remove debris, CD44- cells, aggregates, and dead cells) before submission to the scFlow pipeline.

### Antibodies

Antibodies and their sources used in the canine and feline immunophenotyping panels are detailed in Tables 1, 2, respectively.

**Table 1:** Canine immunophenotyping panel.

**Canine immunophenotyping panel**					
**Marker**	**Clone**	**Fluorophore**	**Vendor**	**Catalog number**	**References**
CD11b	M1/70	BUV395	BD Biosciences	563553	(9)
CCR4	1G1	BUV661	BD Biosciences	752524	(10)
CD49d	9F10	BUV737	BD Biosciences	612850	(11)
CD44	IM7	BUV805	BD Biosciences	741921	(12)
MHCII	CVS20	AF405	Novus Biologicals	NBP2-348-48AF405	(13)
CLA	HECA-452	BV605	BD Biosciences	563960	See nxn plots in Supplementary material
CD62L	SK11	BV786	BD Biosciences	565312	(14)
Neutrophil	CAD048A	AF488	Kingfisher	WS0811D-100	N/A
CD21	CA2.1D6	NovaBlue610-70S	BioRad	MCA1781R	N/A
CD14	M5E2	BB700	BD Biosciences	745790	(14)
CD8a	YCATE55.9	PerCP-eFluor710	Invitrogen	46-5080-42	N/A
CD25	P4A10	PE	BioRad	MCA5916PE	N/A
CD5	YKIX322.3	AF594	BioRad	MCA1037GA	N/A
CD4	296712	AF700	Novus Biologicals	FAB2410N-100UG	N/A
Viability		Zombie NIR	BioLegend	423105	N/A

**Table 2:** Feline immunophenotyping panel.

**Feline immunophenotyping panel**					
**Marker**	**Clone**	**Fluorophore**	**Vendor**	**Catalog number**	**References**
CD49d	9F10	BUV737	BD Biosciences	612850	See nxn plots in Supplementary material; Reported reactivity in cat^a^
CD44	IM7	BUV805	BD Biosciences	741921	See nxn plots in Supplementary material; Reported reactivity in cat^b^
MHCII	CVS20	AF405	Novus Biologicals	NBP2-348-48AF405	See nxn plots in Supplementary material
CLA	HECA-452	BV605	BD Biosciences	563960	See nxn plots in Supplementary material
CD56	HCD56	BV711	BioLegend	318336	(15)
CD62L (SK11)	SK11	BV786	BD Biosciences	565312	(16)
CD4	3-4F4	FITC	Invitrogen	MA5-28776	N/A
Viability		LiveorDye510-550	Biotium	32012	N/A
CD21	CA2.1D6	NovaBlue610-70S	BioRad	MCA1781R	(17)
CD8	fCD8	PE	Southern Biotech	8120-09	N/A
CD14	Tuk4	APC	Invitrogen	MHCD1405	(18)
CD5	f43	Biotin	Southern Biotech	8100-08	N/A
Streptavidin		APC-R700	BD	565144	N/A

^a^https://www.biolegend.com/en-us/products/purified-anti-human-cd49d-antibody-586.

^b^https://www.biolegend.com/en-us/products/purified-anti-mouse-human-cd44-antibody-318.

### Flow cytometry pipeline—scFlow

To support these multispectral flow cytometry analyses, we developed an internal, proprietary full-stack web application, scFlow, featuring a user interface and python backend, whose purpose is to decrease the time spent preprocessing, clustering, and running statistical analyses for flow cytometry data. The scFlow pipeline helps to reduce user bias by standardizing certain aspects of the analysis and using unsupervised machine learning for cell type identification over manual gating. scFlow features six primary steps: preprocessing, aligning and thresholding, batch correction, clustering, integration, and analysis. All software used to build scFlow is publicly available, and packages/parameters are outlined in detail in Supplementary Table 1.

### Preprocessing

The scFlow pipeline begins with reading in raw .fcs file outputs from FlowJo^TM^ Software and then performing a logicle (negative biexponential) transform (*W* = 0.5, *M* = 4.5, *A* = 0) of the raw fluorescence values using the python library FlowKit (v1.0.1). This transformation's purpose is to have logarithmic scaling effect on the exponential scale of the fluorescence intensity across the various markers while handling negative and zero values.

### Alignment

**Figure 1:**
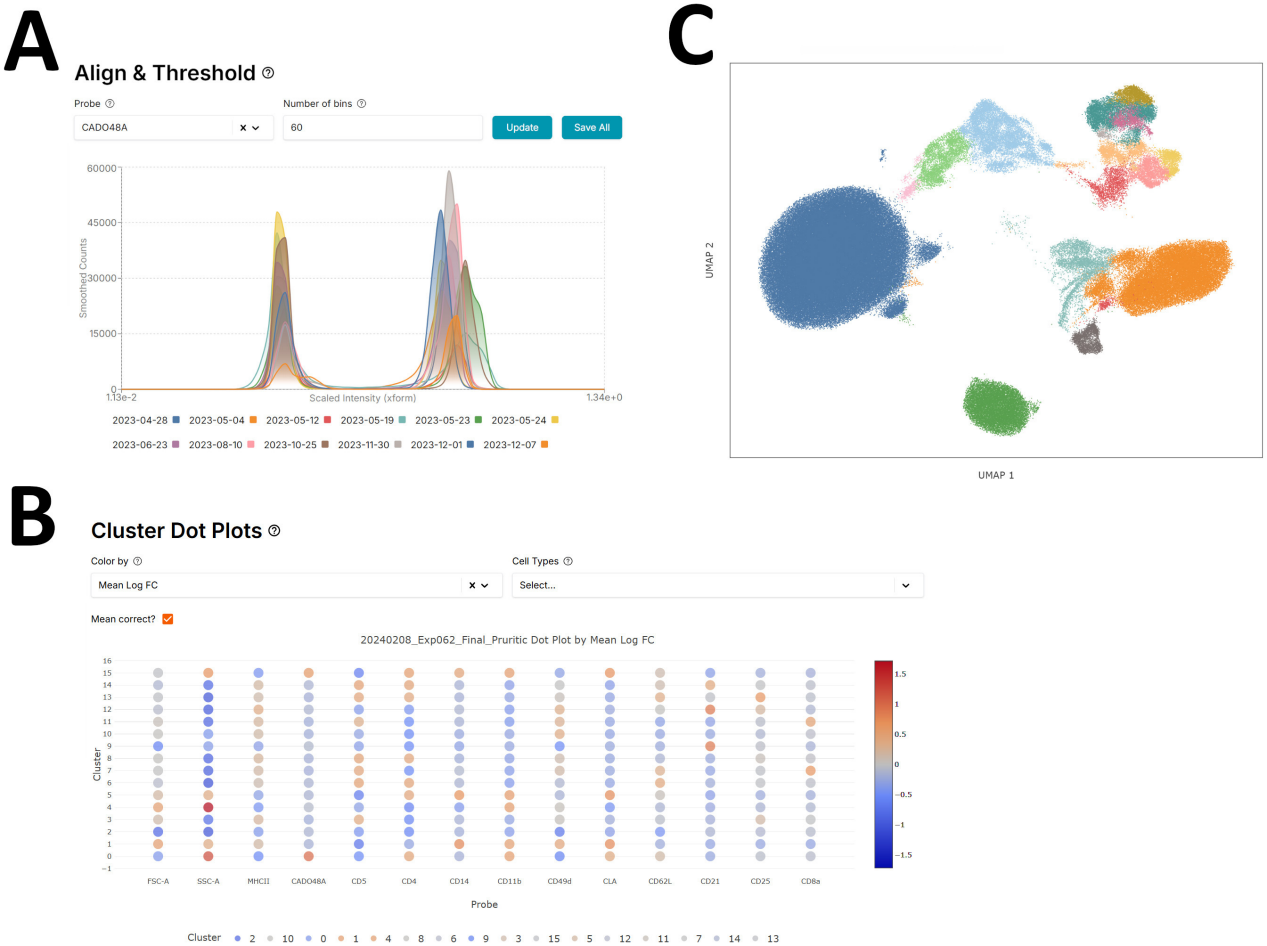
Flow cytometry pipeline - scFlow. **(A)** Aligning negative (early) peaks in different batch distributions for the CADO48A marker using the scFlow app. **(B)** scFlow cluster-marker expression dot plots to identify low (blue) and high (red) marker expression clusters. **(C)** UMAP of cell marker expression colored by manually annotated cell type.

Once samples are transformed, the negative peaks of sample batch distributions for each marker are aligned by either a fluorescence-minus-one (FMO) control or by visual inspection of the negative peaks. This is done as a first-pass manual batch correction where it is assumed that negative marker peaks should align across different batches of samples (Figure 1A).

### Batch correction

After the manual alignment and prior to clustering, we down-sample the cells to 200,000 cells while balancing the number of cells per batch to save computational resources. Then the samples are batch corrected using the Harmony algorithm via the harmonypy python package (v0.0.9) with default parameters. Originally Harmony was designed for single-cell RNA sequencing data, it allows for multiple experimental and biological factors while outperforming previously published batch correction algorithms (19). Harmony was chosen over other methods, such as ComBat, by comparing batch integration across many of our flow cytometry experiments using the first several principal and UMAP components (20, 21). The primary downstream goal of batch correction is to identify cohesive cell-type populations and therefore the minimal batch size (sample) was chosen to produce homogeneous clusters.

### Clustering

Once samples have been batch corrected, then cell clusters are identified using unsupervised clustering on the multi-dimensional marker expression profiles for each cell. This approach has many benefits over the manual gating process traditionally used in the FlowJo software. One main benefit is that such an algorithm uses all the markers at once to determine a non-linear cluster boundary, as opposed to many two-dimensional bounding rectangles, which fail to represent the true cluster boundaries—as well as introduce user bias at each gating step.

scFlow utilizes the Leiden clustering by default via the leidenalg python package (v0.10.1), an iterative hierarchical network-based community detection machine learning algorithm that improves upon the Louvain algorithm (22). At each step, it reassigns subcommunity memberships to optimize community modularity, enhancing cluster quality, see Figure 1B. Users can also further subcluster their clusters to expose rarer cell populations. scFlow boosts computational efficiency by calculating the global neighborhood graph once during the initial clustering phase, then extracting and normalizing each cluster's subgraph before applying Leiden clustering again. Newly identified subclusters are then integrated back into the global community structure, saving time and resources. All parameters outside of the cluster resolution for the leiden algorithm were held constant for this analysis. The value of the resolution changes per clustering/subclustering step to produce more or less clusters. Clusters which are equivalently annotated are consolidated.

scFlow uses cluster-marker expression dot plots to help with annotating cell clusters with a specific cell type, see Figure 1C. The plot features various heatmap settings to better distinguish marker relevance such as the mean intensity or log-fold change difference in marker expression of one cluster vs. the others.

### Integration

After the down-sampled cells have been properly annotated, the out-of-sample cells need to be re-integrated and annotated. scFlow achieves this by computing the entire neighborhood graph across all cells using a naive *k*-nearest neighbors implementation (*k* = 20) leveraging the high-speed and efficient Faiss vector search and indexing library (v1.7.4) developed by Meta (23). This enables the practical and time efficient computation of an entire neighborhood graph containing upwards of tens of millions of cells.

Once the graph has been computed, an out-of-sample cell can be annotated by looking at the cell types of its annotated neighbors and taking a majority vote. scFlow allows a confidence percentage threshold *X* to be set so that any out-of-sample cells which have less than *X%* neighbors of the majority cell type are discarded from downstream analysis. This allows the user to have high-confidence in the machine-assigned cell type. A confidence percentage threshold of *X* = 80% was used for the results of this paper. The user can also get a breakdown of the percent mapped per cell type to identify any transient cell populations, i.e., those populations which are blended with many other well-established cell types.

### Analysis

Two non-parametric statistical tests are used for evaluating differences between two comparison groups. The first test, the Wilcoxon Rank Sum (WRS) Test (also known as the Mann Whitney *U*-Test), utilizes the sum of the ranks to determine if the groups come from the same distribution. The second test, the two-sample Kolmogorov-Smirnov (KS) test, employs the largest difference between two distributions as a test statistic to assess if the distributions are identical. The KS test's sensitivity increases with sample size, making it particularly suitable for analyzing flow cytometry data, which can involve millions of cells.

Both statistics generate *p*-values and *q*-values (false discovery rate, FDR) to order the comparisons by. The *q*-values in all cases are computed via the Benjamini-Hochberg (BH) procedure, which considers all the *p*-values across the different comparisons and corrects each one relative to its significance to the rest. While the Bonferroni correction controls the family-wise error rate by adjusting the significance threshold, the BH procedure is less conservative and controls the false discovery rate, allowing for a higher rate of true positive findings, a desired feature within discovery.

In the cell proportion analysis, the proportion of each cell type is computed per sample, and the resulting distributions across samples for each group and cell type are compared. Both WRS and KS are used to determine the statistical significance of cell proportion distribution differences. See the Supplementary Table 1 for all python packages and their specific versions used for this analysis.

## Results

### Canine hematological data

**Figure 2:**
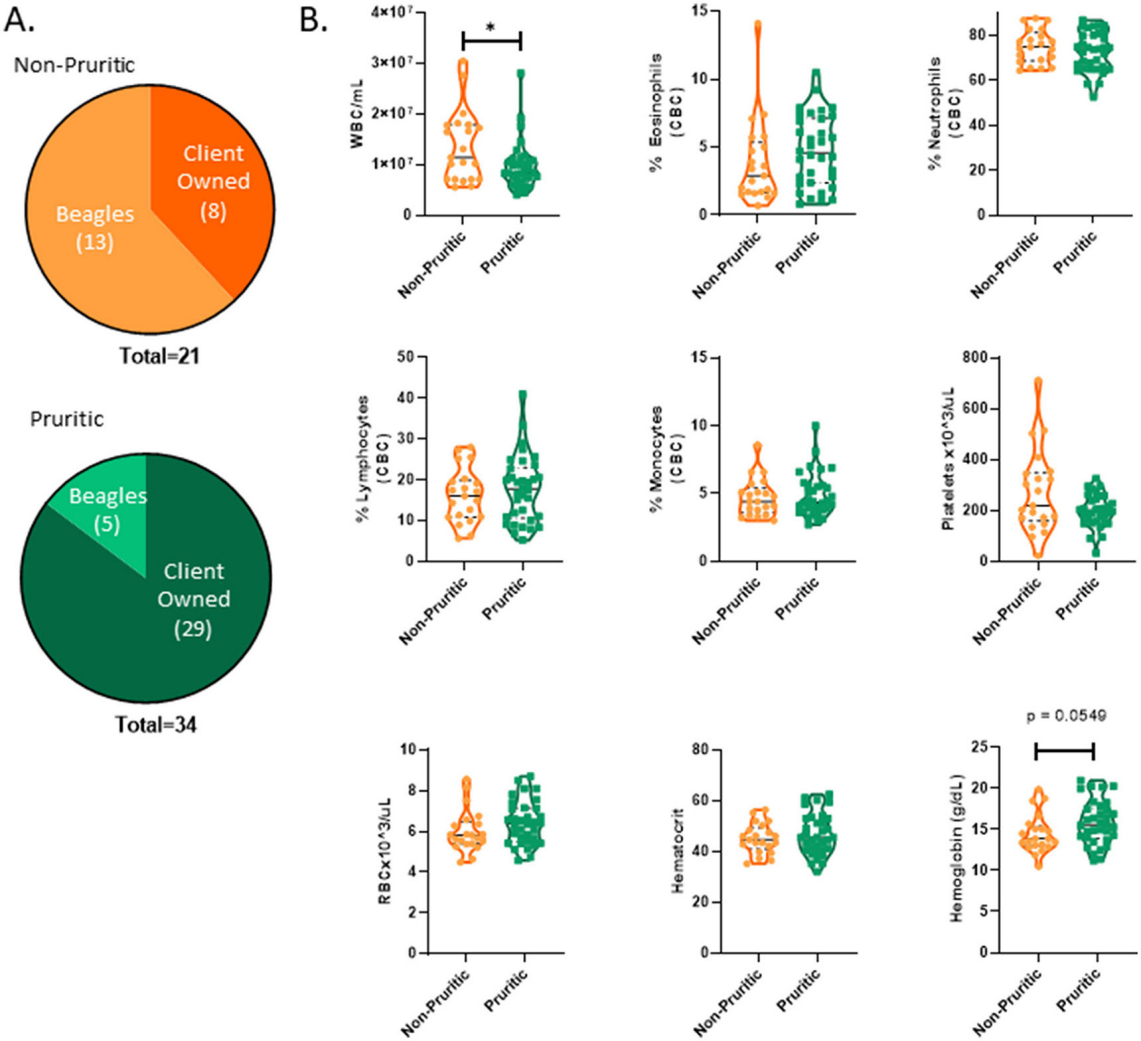
Pruritic dogs have significantly decreased White Blood Cell (WBC) counts compared to non-pruritic dogs. Breakdown of pruritic and non-pruritic dogs **(A)**. Blood collected in K2EDTA tubes were inverted multiple times and then were run on the Element HT5 automated hematology analyzer (Heska Corporation, USA) for complete blood cell (CBC) counts and a five-part white blood cell differential **(B)**: WBCs, Eosinophils, Neutrophils, Lymphocytes, Monocytes, Platelets, RBCs, Hematocrit, Hemoglobin. JMP was used for statistical analyses and plots were made in Graphpad. Violin plots were used to visualize the distribution of data [*n* = 21 non-pruritic dogs (orange) *n* = 34 pruritic dogs (green)]. Statistical analysis was performed by unpaired t test with Welch's correction. *Two-tailed *p*-value = 0.0313.

A breakdown of the sources of pruritic and non-pruritic dogs is shown in Figure 2A. Table 3 summarizes the canine hematological data. While pruritic dogs had significantly (*p* = 0.0313) decreased white blood cell (WBC) counts than non-pruritic dogs, there were no significant differences in the percentage of circulating neutrophils, monocytes, lymphocytes, or eosinophils. Platelets were slightly decreased (*ns*) in pruritic dogs compared to non-pruritic dogs. Although not significant, pruritic dogs had an increase in hemoglobin concentration compared to non-pruritic dogs (*p* = 0.0549), despite no significant differences noted in RBC counts (or hematocrit values) (Figure 2B) (see Table 3).

**Table 3:** Canine hematological data.

**Parameter (±Std dev)**	**Non-pruritic dogs (21)**	**Pruritic dogs (34)**	**Reference range (Heska Element HT5)**	***p-*value**
WBC (× 10^3^/μL)	13.6 ± 7.1	9.6 ± 4.6	6–17	0.0313^*^
EOS (× 10^3^/μL)	0.45 ± 0.30	0.47 ± 0.41	0.04–1.62	0.8521
LYM (× 10^3^/μL)	1.9 ± 0.8	1.5 ± 0.6	0.83–4.91	0.0717
MONO (× 10^3^/μL)	0.6 ± 0.3	0.5 ± 0.3	0.14–1.97	0.0952
NEU (× 10^3^/μL)	10.5 ± 6.3	7.3 ± 4.0	3.62–12.3	0.0428^*^
EOS %	3.8 ± 3.1	4.7 ± 2.6	0.5–10	0.2594
LYM %	16.1 ± 6.6	17.7 ± 8.2	12–33	0.4461
MONO %	4.7 ± 1.4	4.8 ± 1.6	2–13	0.7849
NEU %	75.2 ± 7.4	72.7 ± 8.7	52–81	0.2645
PLT (× 10^3^/μL)	272.1 ± 164.2	199.6 ± 63.5	117–490	0.0647
RBC (× 10^3^/μL)	6.0 ± 1.0	6.4 ± 1.1	5.1–8.5	0.1773
HCT %	45.2 ± 5.9	46.6 ± 8.0	33–56	0.4331
HGB (g/dL)	14.5 ± 2.4	15.8 ± 2.7	11–19	0.0549

### Canine immunophenotyping

**Figure 3:**
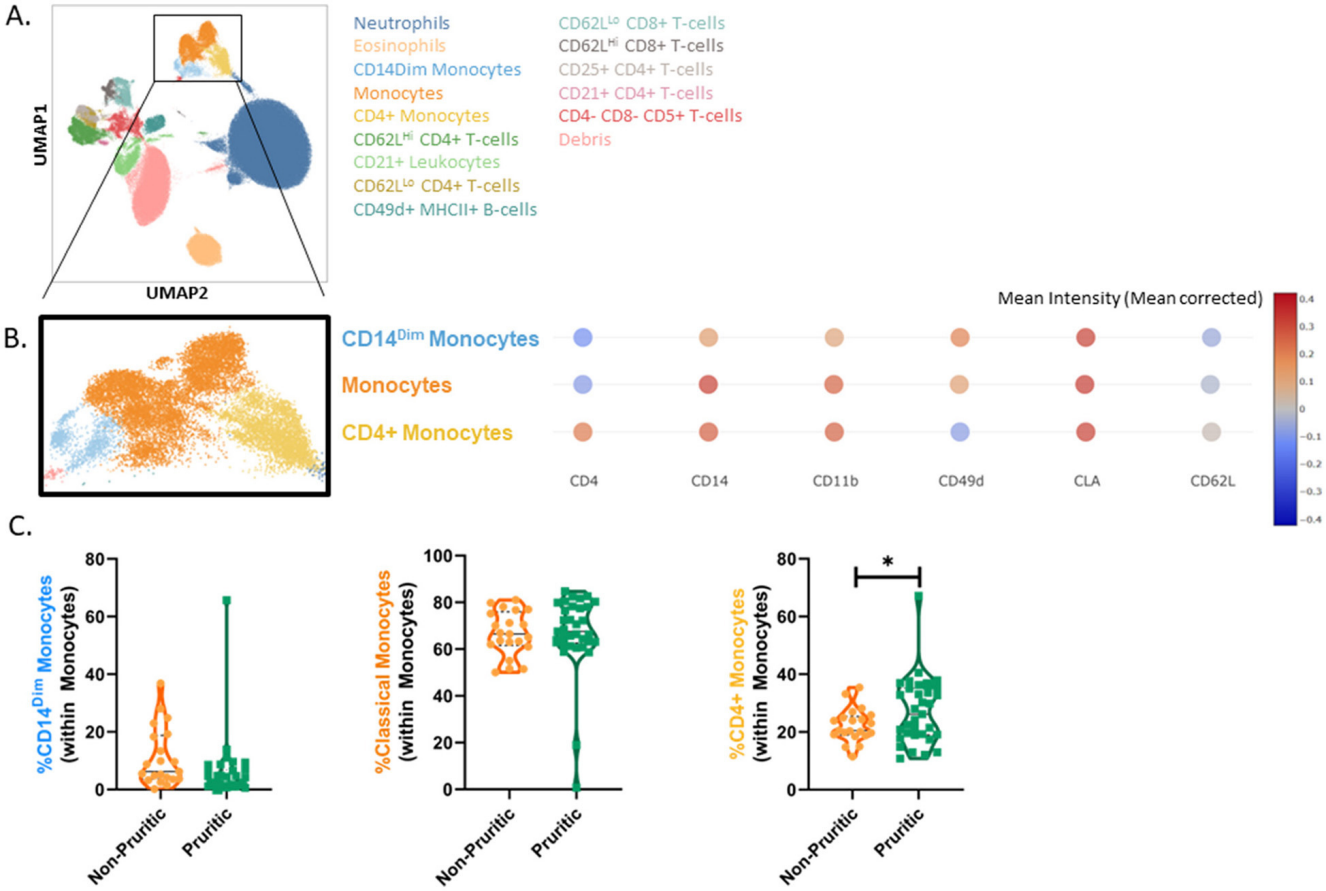
scFlow clustering analysis. Whole blood was stained with Zombie NIR Fixable viability stain and a 14-color immunophenotyping panel. Unique populations were identified through clustering, including subpopulations of CD4+ and CD8+ T-cells and Monocytes **(A)**. Three monocyte subsets were identified in the dog by evaluating the mean intensity (mean corrected) of key markers **(B)** and quantified **(C)**. Data shown are violin plots [*n* = 21 healthy dogs (orange) and *n* = 33 pruritic dogs (green)]. Statistical analysis was performed by unpaired *t*-test with Welch's correction. *Two-tailed *p*-value = 0.0441.

We were able to identify the major cell populations in dogs and cats (neutrophils, eosinophils, monocytes, and lymphocytes) and subclustering showed unique subsets of these populations. All clusters were identified by using expression (or lack of expression) of integrins, cell surface molecules, and known activation markers (Figure 3A). Canine eosinophils were identified by their increased granularity relative to other cell types (high side scatter; SSC), their large size and their inherent autofluorescent nature.

Clustering analysis of the canine flow cytometry data identified three subsets of monocytes: classical monocytes, CD4+ monocytes and CD14^Dim^ monocytes. Expression of CD4, CD14, CD11b, CD49d, CD62L, and MHCII were used to classify the monocytes into subsets (Figure 3B). CD14^Dim^ monocytes had decreased expression of CD14, CD11b, and CD62L compared to classical monocytes. CD4+ monocytes were unique in their expression of CD4; these monocytes also had decreased MHCII expression and CD49d expression relative to the other monocyte subsets. While the fraction of monocytes reported by the CBC differential did not differ between non-pruritic and pruritic dogs, there was a significant increase (*p* = 0.0441) in the fraction of CD4+ monocytes within the monocyte population of pruritic dogs compared to non-pruritic dogs (Figure 3C).

**Figure 4:**
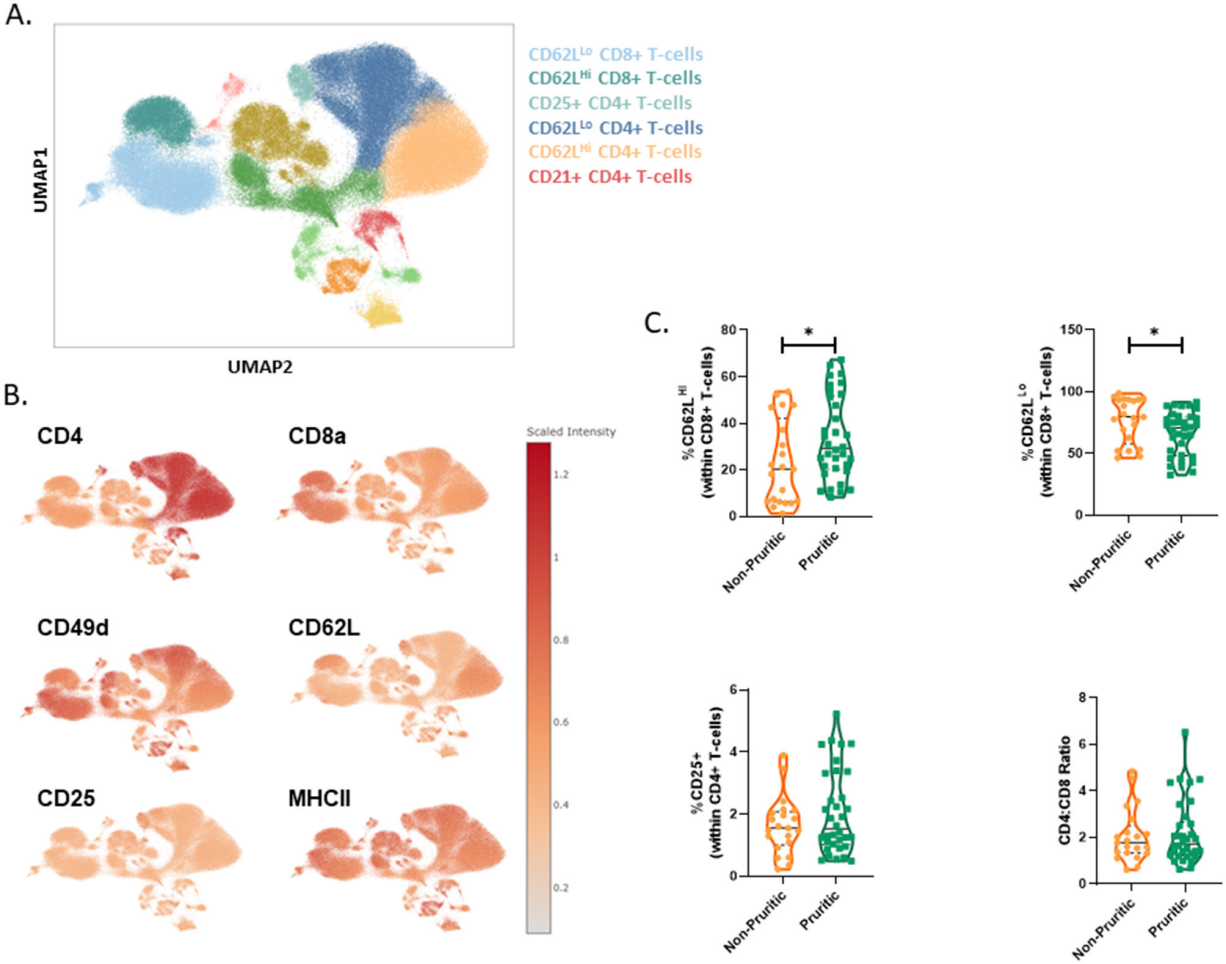
scFlow clustering analysis of CD5+ Lymphocytes. CD5+ Lymphocytes were clustered and T-cell subsets were identified **(A)**. Umaps showing marker expression **(B)**. Quantification of T-cell subsets in dogs **(C)**. Data shown are violin plots [*n* = 21 non-pruritic dogs (orange) and *n* = 33 pruritic dogs (green)]. Statistical analysis was performed by unpaired *t*-test with Welch's correction. *Two-tailed *p*-value = 0.0361.

The CBC differential only reports total lymphocytes, whereas multispectral flow cytometry allows unique identification of T-cells and B-cells. Surface expression of CD21, CD49d and MHCII were used to identify canine B-cells. The fraction of B-cells did not differ between pruritic or non-pruritic dogs. T-cell subsets were identified in the original clustering, but to better define T-cell populations, CD5+ lymphocytes were gated and the CD5+ population was clustered separately (Figure 4A). Expression levels of CD62L, CD49d and CD25 were used to define T-cell subsets (Figure 4B). The fraction of CD62LHi CD8+ T-cells was increased in pruritic dogs. There was a trend for an increased fraction of CD25+ CD4+ T-cells. There was no difference in the CD4:CD8 T-cell ratio between pruritic or non-pruritic dogs (Figure 4C).

### Feline hematological data

**Figure 5:**
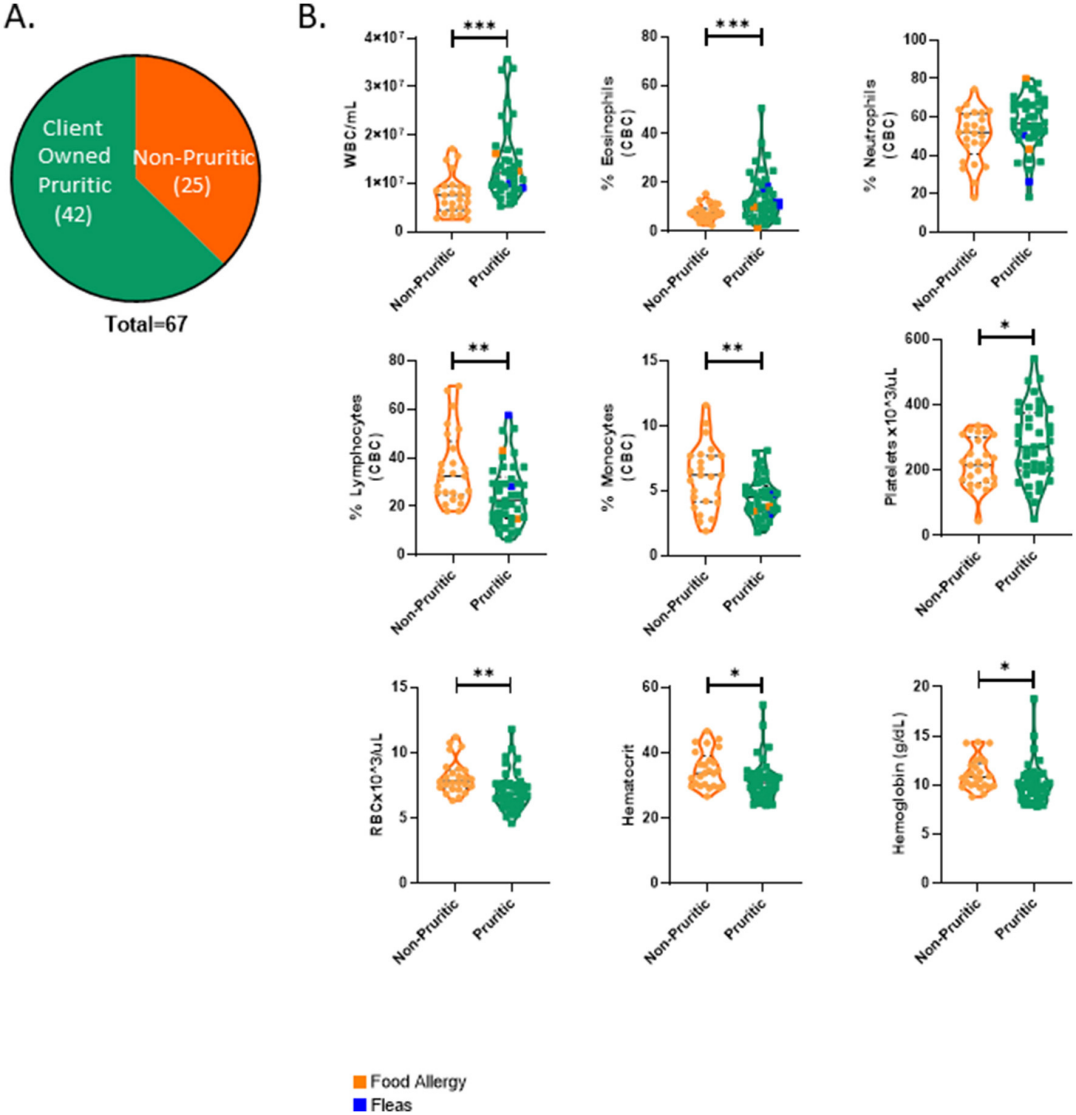
Pruritic cats differ significantly from non-pruritic cats in multiple hematological parameters. Breakdown of pruritic and non-pruritic cats **(A)**. Blood collected in K2EDTA tubes were inverted multiple times and then were run on the Element HT5 automated hematology analyzer (Heska Corporation, USA) for complete blood cell (CBC) counts and a five-part white blood cell differential **(B)**: WBCs, Eosinophils, Neutrophils, Lymphocytes, Monocytes, Platelets, RBCs, Hematocrit, Hemoglobin. JMP was used for statistical analyses and plots were made in Graphpad. Violin plots were used to visualize the distribution of data [*n* = 25 non-pruritic cats (orange); n = 42 pruritic cats (green); within the pruritic cats, cats that had food allergy are colored dark orange and cats with known fleas are colored in blue]. Statistical analysis was performed by unpaired *t*-test with Welch's correction. Two-tailed *p*-values are reported: *p < 0.05, **p < 0.01, **p < 0.0001.

A breakdown of the number of pruritic and non-pruritic cats is shown in Figure 5A. Table 4 summarizes the feline hematological data. The WBC count was significantly increased (*p* < 0.0001) in pruritic cats compared to non-pruritic cats and pruritic cats had significant shifts in circulating immune cells (Figure 5B). The absolute counts of eosinophils and neutrophils were significantly increased in pruritic cats (*p* < 0.0001) as compared to non-pruritic cats. See Table 4 for complete feline hematological results.

**Table 4:** Feline hematological data.

**Parameter (±Std dev)**	**Non-pruritic cats (25)**	**Pruritic cats (42)**	**Reference range (Heska Element HT5)**	***p-*value**
WBC (× 10^3^/μL)	7.7 ± 4.0	14.2 ± 7.6	5.5–19.5	< 0.0001^*^
EOS (× 10^3^/μL)	0.56 ± 0.30	2.0 ± 1.8	0.06–1.93	< 0.0001^*^
LYM (× 10^3^/μL)	2.7 ± 1.7	3.3 ± 2.2	0.73–7.86	0.1792
MONO (× 10^3^/μL)	0.47 ± 0.30	0.67 ± 0.56	0.07–1.36	0.0657
NEU (× 10^3^/μL)	4.0 ± 2.6	8.2 ± 5.0	3.12–12.58	< 0.0001^*^
EOS %	7.8 ± 3.2	14.1 ± 10.0	1–11	0.0005^*^
LYM %	35.9 ± 15.2	25.1 ± 12.5	12–45	0.0044^*^
MONO %	6.1 ± 2.5	4.5 ± 1.6	1–8	0.0063^*^
NEU %	50.2 ± 13.6	56.3 ± 13.9	38–80	0.0808
PLT (× 10^3^/μL)	220.0 ± 74.3	280.4 ± 112.1	100–518	0.0101^*^
RBC (× 10^3^/μL)	8.17 ± 1.30	7.07 ± 1.46	4.6–10.2	0.0023^*^
HCT %	34.93 ± 5.58	31.41 ± 6.17	26–47	0.0198^*^
HGB (g/dL)	11.14 ± 1.58	10.14 ± 2.06	8.5–15.3	0.0300^*^

### Feline immunophenotyping

**Figure 6:**
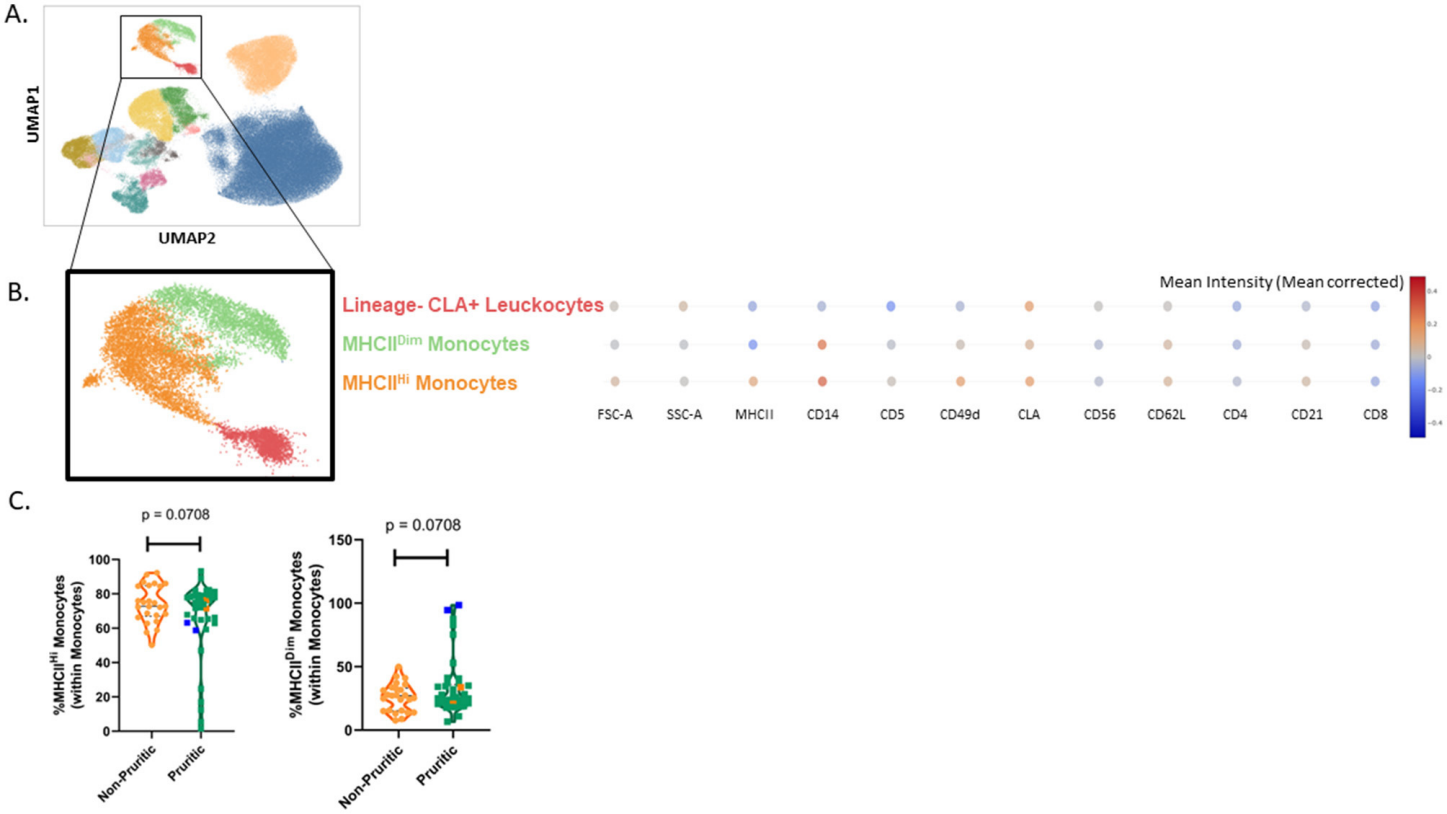
Cat scFlow clustering analysis. Whole blood was stained with Live or Dye 510 viability stain and an 11-color immunophenotyping panel. Unique populations were identified through clustering, including subpopulations of CD4+ and CD8+ T-cells and Monocytes **(A)**. Two unique monocyte subsets were identified in the cat by evaluating the mean intensity (mean corrected) of key markers **(B)** and quantified **(C)**. Violin plots were used to visualize the distribution of data [*n* = 25 non-pruritic cats (orange); *n* = 42 pruritic cats (green); within the pruritic cats, cats that had food allergy are colored dark orange and cats with known fleas are colored in blue]. Statistical analysis was performed by unpaired *t*-test with Welch's correction. Two-tailed *p*-values are reported.

As described for the canine immunophenotyping results, our analysis showed separate clusters for the major immune cell populations (neutrophils, eosinophils, monocytes, and lymphocytes). These clusters were further subclustered and identified by expression (or lack of expression) of integrins, cell surface molecules, and known activation markers (Figure 6A). Like canine eosinophils, feline eosinophils have increased granularity relative to other cell types, are much larger than other cell types, and are inherently autofluorescent. As reported in the CBC, scFlow analysis also identified eosinophils as being significantly increased (*p* = 0.0019) in the pruritic cats compared to non-pruritic cats (Supplementary Figure 1). We also identified two monocyte subclusters in the cat: MHCII^Hi^ monocytes and MHCII^Dim^ monocytes (Figure 6B). Both monocyte subsets expressed CD49d and cutaneous lymphocyte-associated antigen (CLA), although the overall expression of both markers was decreased in the MHCII^Dim^ population. There was a unique leukocyte population that clustered near the monocytes, but it was dim for both CD14 and MHCII expression (Lineage- CLA+ Leukocytes); this population was not annotated as a monocyte subset. When total monocytes (MHCII^Hi^ subset combined with MHCII^Dim^ subset) were evaluated, the majority of monocytes were MHCII^Hi^ (Figure 6C).

**Figure 7:**
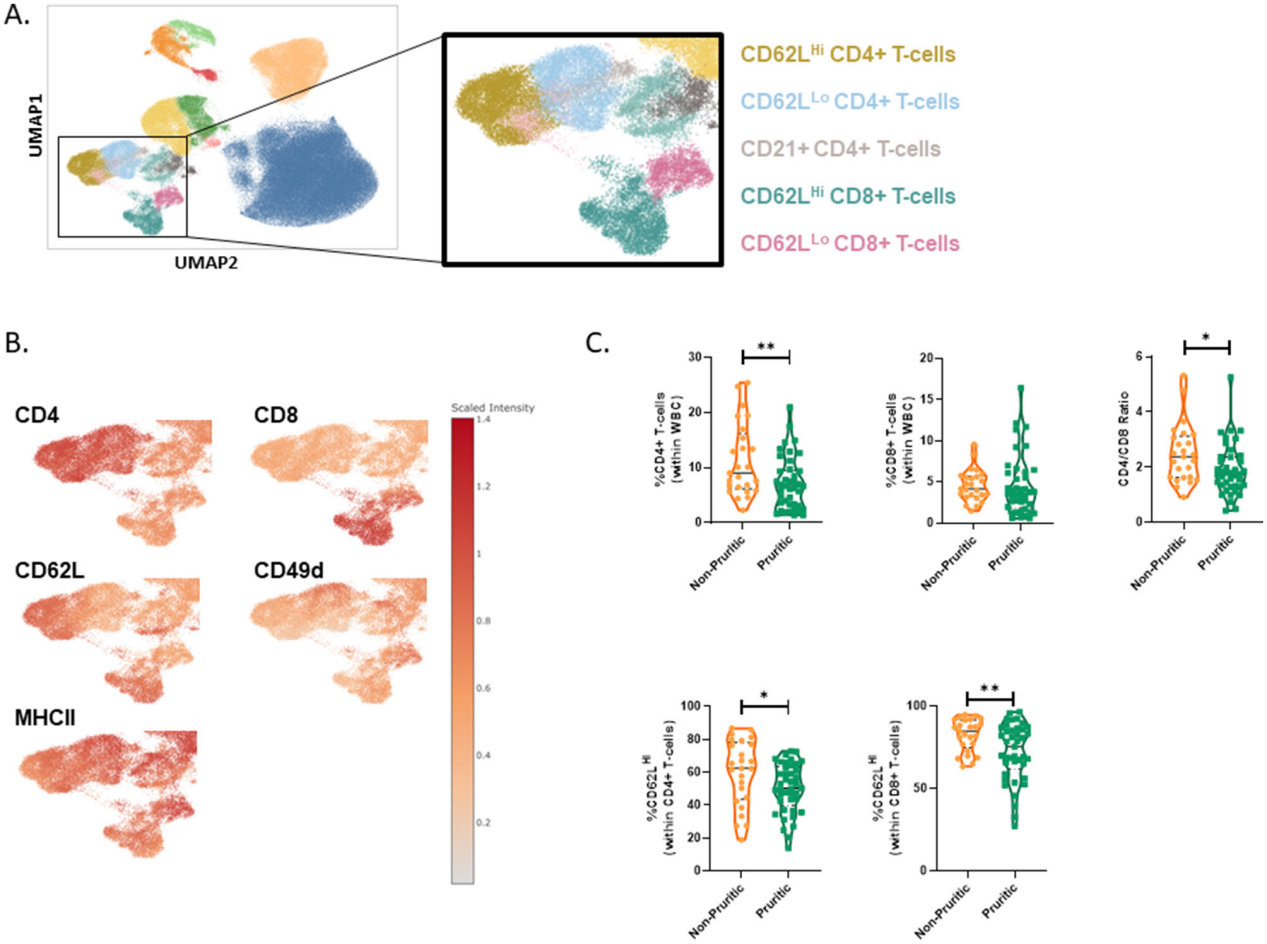
T-cells in cats. T-cell subsets were identified **(A)**. Umaps showing marker expression **(B)**. Quantification of T-cell subsets in cat **(C)**. Violin plots were used to visualize the distribution of data [*n* = 25 non-pruritic cats (orange); *n* = 42 pruritic cats (green); within the pruritic cats, cats that had food allergy are colored dark orange and cats with known fleas are colored in blue]. Statistical analysis was performed by unpaired *t*-test with Welch's correction. Two-tailed *p*-values are reported: CD4+ T-cells (within WBC) **p = 0.0083; CD4/CD8 ratio *p = 0.0255; CD62L^Hi^ (within CD4+ T-cells) *p = 0.0435; CD62L^Hi^ (within CD8+ T-cells) **p = 0.0028.

CD4+ and CD8+ T-cell subsets were further subdivided into CD62L^Lo^ vs CD62L^Hi^ subsets (Figure 7A). The CD62L^Hi^ T-cell subsets had decreased expression of CD49d relative to the CD62L^Lo^ subsets (Figure 7B). Overall, there were fewer circulating total CD4+ T-cells in pruritic cats compared to non-pruritic controls (Figure 7C). Within the T-cell populations, there was a significantly decreased fraction of CD62L^Hi^ CD4+ T-cells (within the CD4+ T-cell population) and CD62L^Hi^ CD8+ T-cells (within the CD8+ T-cell population) in pruritic vs. non-pruritic cats (*p* = 0.0435 and *p* = 0.0028, respectively, Figure 7C). Lastly, pruritic cats had an overall significantly decreased (*p* = 0.0255) CD4+ T-cell:CD8+ T-cell ratio compared to non-pruritic cats (Figure 7C).

**Figure 8:**
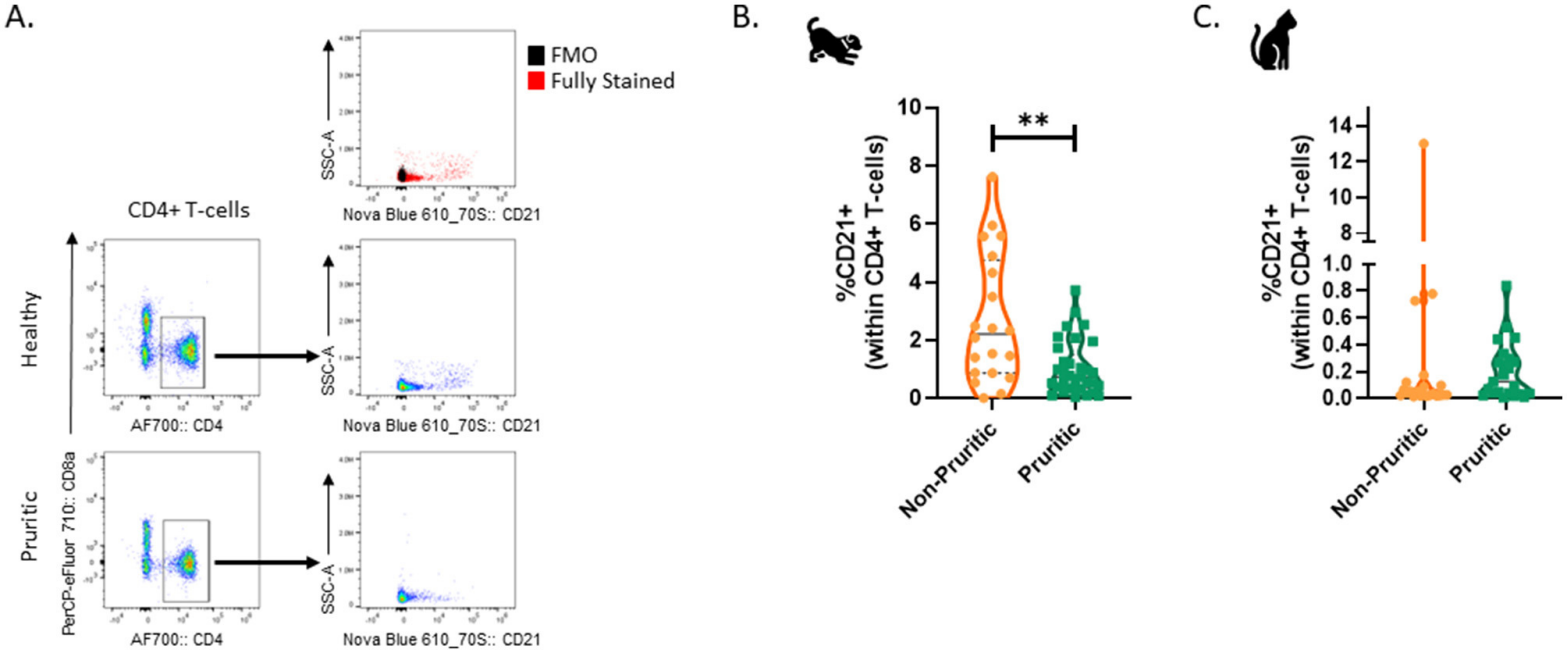
CD21+ CD4+ T-cells in dogs and cats. Plots based on previously gated cells; Cells were first gated to exclude debris and CD44- cells, aggregates, and dead cells. CD5+ cells were then gated, and these CD5+ cells were analyzed for CD4+ or CD8+ expression **(A)**. The % of CD21+ cells within the CD4+ T-cell population were quantified in dogs **(B)** and cats **(C)**. Violin plots were used to visualize the distribution of data (non-pruritic animals (orange) and pruritic animals (greens). Statistical analysis was performed by unpaired *t*-test with Welch's correction. Two-tailed *p*-values are reported: **p = 0.0036.

Lastly, a CD21+ CD4+ T-cell population was identified in the CD5+ leukocytes within both dogs and cats. A representative flow cytometry plot showing CD21+ CD4+ CD5+ T-cells is shown in Figure 8A. The fraction of CD21+ cells within CD4+ T-cells was significantly decreased (*p* = 0.0036) in pruritic dogs vs. non-pruritic dogs (Figure 8B). This population had no difference between pruritic cats vs. non-pruritic cats (Figure 8B). This unique CD4+ T-cell population was rare in cats (Figure 8C) (0.7 and 0.2% of CD4+ T-cells in non-pruritic and pruritic cats respectively, vs. 2.7 and 1.0% of CD4+ T-cells in non-pruritic and pruritic dogs).

## Discussion

Using a combination of standard hematological parameters and high parameter immunophenotyping panels, we achieved a detailed characterization of circulating immune cell populations in dogs and cats. Our immunophenotyping panels allowed us to identify cell populations analogous to those in humans and mice, and to uncover species-specific subsets. By focusing on pruritis as a clinical presentation, we identified significant differences in immune cell populations between non-pruritic and pruritic animals. While pruritus is a key indicator of allergic and atopic dermatitis, other differential diagnoses must be ruled out. Thus, this study does not claim causation, but rather highlights the need for further rigorous studies to understand the role of altered immune cell proportion in pruritic cats and dogs and their potential impact on dermatological disorders.

Total WBC counts were significantly decreased in pruritic dogs compared to non-pruritic dogs. The statistically significant decrease in WBC in pruritic dogs was due to a slight decrease in the neutrophil count, without impact on the leukocyte differential. However, it should be considered that many different variables such as breed, age, underlying disease pathophysiology, chronicity of disease, comorbidities, and concomitant treatments, were not considered in the study and may have affected CBC. For example, glucocorticoids cause alterations in the leukogram such as an increase in circulating neutrophils and monocytes and a decrease in overall lymphocyte populations (24). Due to the limited number of animals in the study, dogs are of mixed breeds, different ages, may be receiving concomitant treatments and have comorbidities that were not assessed.

Since pruritis is a cardinal sign of canine Atopic Dermatitis (cAD), we used cAD literature to provide context for our findings. Previous reports on the blood lymphocyte population in diagnosed cAD cases have shown an overall decrease in lymphocytes (25, 26). Martins et al. show a specific increase in CD4+ T-cells and a decrease in CD8+ T-cells in dogs diagnosed with atopic dermatitis. This could be due to potential therapeutics either group is receiving or perhaps that changes in these parameters are not strong indicators of atopic dermatitis. In a study that profiled T-cells in dogs treated for adverse food reactions (clinical signs assessed with a pruritis Visual Analog Scale) with food allergen-specific sublingual immunotherapy, treated dogs had a decrease in CD8+ T-cells (27). CD4+ and CD8+ T-cell subsets were either CD62L^Hi^, indicative of either a more naive-like phenotype or a memory phenotype, or CD62L^Lo^, which is more characteristic of effector T-cells (2). CD62L expression has been well-studied in humans and mice, and in the context of this paper we are using humans/mice as models for dogs and cats. In our current study, CD62L^Hi^ T-cells within the parent CD8+ T-cell population were significantly increased in pruritic vs. non-pruritic dogs. Naïve, newly activated, and expanded, and central memory T-cells are CD62L^Hi^ and circulate between blood, lymph, and secondary lymphoid organs (2). The increase in CD62L^Hi^ CD8+ T-cells could imply a clonal expansion of newly activated CD8+ T-cells in pruritic dogs. Further immunophenotyping is required to further subset these T-cells and provide insight into function; CD49d (integrin α4) binds the ligands VCAM-1 and fibronectin and facilitates lymphocyte extravasation (28, 29). In humans and mice, memory T-cells are characterized by elevated expression levels of CD49d, relative to naïve T-cells (30–32). Whether this is true for canine memory T-cells remains to be discovered.

Regulatory T cells (Tregs) are an immunomodulatory T cell subset that has been implicated in human AD (33, 34). However, published studies assessing circulating Tregs in dogs diagnosed with cAD have not shown consistent differences in this cell population. One study found that circulating Tregs (identified as CD4+CD25+FoxP3+) were significantly increased in dogs diagnosed with cAD compared to healthy controls (35), while one other study showed elevated (not significant). Tregs and a third study showed no difference in Tregs, between untreated cAD dogs and non-pruritic healthy controls (36, 37). This may be due to differences in reporting of Treg percentages (as percent of total peripheral blood leukocytes, or within CD4+, or within CD4+CD25+ cells), or differences in age or disease severity in the analyzed cohorts. A shortcoming of our current study is that it only analyzed surface markers and did not include intracellular staining. Since newly activated effector T cells also express CD25, we cannot differentiate Tregs from newly activated effector T cells in our dataset. We did not observe a statistically significant increase in CD25+ cells within the circulating CD4+ population.

Due to limited fluorophore availability, an anti-canine TCRγδ was not included in the immunophenotyping panel. Future improvements should incorporate a marker for γδ T-cells. Psoriasis and atopic dermatitis in humans, conditions characterized by severe itching, have been linked to IL-17/IL-22 producing γδ T-cells (38, 39). Additionally, a recent study by Sparling et al. used single-cell transcriptomics to identify differences between healthy control and AD skin in canines, revealing an enrichment of γδ T-cells in AD skin (40).

The hematological profile of pruritic cats was different than what was observed in pruritic dogs. Pruritic cats had leukocytosis compared to non-pruritic cats. Both CBC and flow cytometry demonstrated that eosinophils were significantly increased in pruritic vs. non-pruritic cats. Whether eosinophils play a significant role in initiating or perpetuating itch in cats remains to be seen. In a study of clinical human AD (Atopic Dermatitis) patients, eosinophil count correlated with the frequency of circulating TSLPR+ CD4+ T-cells (41).

Despite only a relative variation (decrease) of lymphocytes in the CBC, immunophenotyping revealed a shift in T-cell populations. CD62L^Lo^ T-cells, which are activated or effector memory T-cells (2), showed a significant decrease in CD62L^Hi^ CD4+ T-cells and CD8+ T-cells (within their respective populations) in the pruritic cats compared to non-pruritic cats. This suggests a shift toward activated T-cells and/or effector memory T-cells, which can access non-lymphoid tissues such as the skin. Previous histopathological examination of lesional and non-lesional skin from cats with allergic dermatitis, as well as skin from non-pruritic controls, revealed an immune cell infiltration of CD4+ and CD8+ T-cells in the skin. Additionally, there were significantly more IL-4+ T-cells, in cats with allergic dermatitis compared to non-pruritic control cats (42, 43). In atopy patch testing (APT) of cats with spontaneously occurring atopic dermatitis, an increase of IL-4+ T-cells infiltrating into the APT biopsy site was also observed (42). It would be interesting to characterize CD62L expression levels on these IL-4+ T-cells.

Unsupervised clustering identified a unique CD21+ CD4+ T-cell population within the CD5+ leukocytes for both dogs and cats, which was verified by manual gating. CD21, also known as complement receptor 2, is typically expressed on B-cells and follicular dendritic cells (44, 45). Epstein Barr Virus (EBV), a human pathogen, uses CD21 as a receptor to infect B-cells (46). Although serological evidence indicates exposure to EBV or an EBV-like virus in dogs and cats via detection of antibodies to EBV antigens in serum, no infectious virus has been detected (47, 48). CD21+ CD4+ T-cells were rare, comprising only ~1% of CD4+ T-cells. We speculate that the expression of complement receptor 2 (CD21) on T-cells may provide an additional mechanism for T-cells to regulate complement activation. Recently, Riondato et al. identified CD5+ CD21+ dim lymphocytes in canine lymph nodes (49). Alternatively, these T-cells might have bound soluble CD21 or be in the process of shedding soluble CD21. In 1998, it was reported that human B and T-lymphocytes spontaneously released soluble CD21 (50).

Monocyte subsets in both the cat and dog were identified by unsupervised clustering. These subsets were driven by differences in CD49d, MHCII, CLA, and CD14 expression. In humans and many other well-studied species, multiple subsets of monocytes exist and are differentiated by specific markers and functions, for example CD14 and CD16 (humans) and Ly6C (mice) (51). Feline monocytes are poorly characterized phenotypically and to date very few transcriptomic studies have been published (52–54). Two feline monocyte subsets were found by unsupervised clustering and verified by manual gating: MHCII^Hi^ monocytes and MHCII^Dim^ monocytes. Pruritic cats had a significant decrease in the fraction of monocytes relative to non-pruritic cats, and this was driven by a decrease in MHCII^HI^ monocytes. One hypothesis is that the increased expression of integrins CLA and CD49d on these monocytes might drive their migration into tissues, thus leaving less circulating in the blood. Other cell types increased in feline skin lesions are MHCII class II+ epidermal dendritic cells, CD1a+ Langerhans cells, macrophages, and dermal mast cells (55, 56). Whether these macrophages and/or dendritic cells come from monocyte precursors circulating in the blood is unknown. More work needs to be done to functionally characterize these monocyte subsets, and it will be critical to assess the expression level of these integrins using quantitative methods.

There is no consensus on canine monocyte subsets, although attempts have been made to characterize them using different antibody panels consisting of unique combinations of CD11b, MHCII, CD14 and CD4 (57, 58). In dogs, we used CD11b, CLA and CD4 to further delineate three monocyte subsets: CD4+ monocytes, CD14^Dim^ monocytes, and classical monocytes. Internal single cell RNAseq data on canine PBMCs has verified these three monocyte subsets (data not shown) and another recent scRNA seq study corroborated our findings (59). CD4, normally associated with identifying CD4+ T-cells, is also a marker of canine myeloid cells, including all neutrophils and a subset of monocytes and dendritic cells (60). The function of CD4 in these myeloid subsets is unknown (58, 61, 62). CD4+ monocytes are not unique to dogs; they are also detected in certain myeloid subsets in humans and rat (63–66). Recent work has shown that ligation of CD4 on human monocytes triggers macrophage differentiation (66). Whether this is true in canine is yet to be discovered.

While the fraction of total monocytes in the dogs did not differ between non-pruritic or pruritic states, pruritic dogs did have a significant increase in CD4+ monocytes within the parent monocyte population. Although CD4+ monocytes have decreased expression of CD49d and MHCII relative to the other monocyte subsets, they do express other integrins important for rolling adhesion, migration, and signaling (such as CD11b, CLA and CD62L). In a separate internal study, circulating CD4+ monocytes also increased in dogs that were undergoing acute gastrointestinal distress (pancreatitis or colitis) at the time of blood draw (data not shown). This subset of monocytes may be reflective of increased inflammation. Their journey from the bone marrow, release into the blood, trafficking into inflamed tissues, and their contribution to disease remains to be studied. Myeloid-derived suppressor cells (MDSCs) have been identified in dogs and are usually identified by their lack of MHCII expression (9, 67, 68). Thus, a small fraction of these CD4+ monocytes may be MDSCs (59). MDSCs are elevated in many acute and chronic inflammatory states, including psoriasis and cancer (69, 70). To truly identify an MDSC, a functional assessment of their ability to suppress proliferation and IFN-γ production in T-cells would have to be carried out.

Overall, our study demonstrates the value of developing well-validated high parameter veterinary immunophenotyping panels. The combination of spectral flow cytometry and an AI-driven clustering data analysis algorithms allow for higher-resolution data collection to better capture and characterize the complexity of circulating immune cell populations and their activation states. Herein, we have identified novel populations of immune cells and demonstrated their altered proportions in pruritic cats and dogs, warranting further studies to understand their role in dermatological disorders. Such hypothesis generating data sets will contribute to the identification of biomarkers, therapeutic targets, and improved understanding of immune cell subsets present in veterinary species and is on par with similar efforts in human medicine.

## Data Availability

The raw data supporting the conclusions of this article will be made available by the authors, without undue reservation.

## Supplementary material

Supplementalry Figure 1 scFlow corroborates CBC data for eosinophils. Violin plots were used to visualize the distribution of data (n = 25 non-pruritic cats (orange); n = 42 pruritic cats (green); within the pruritic cats, cats that had food allergy are colored dark orange and cats with known fleas are colored in blue). Statistical analysis was performed by unpaired t test with Welch's correction. Two-tailed p-values are reported: ^***^p = 0.0005; ^**^p = 0.0019.

Supplementary Figure 2 Canine gating strategy to gate out debris and CD44- cells, aggregates, and dead cells **(A)**. Dot plots of SSC-A vs each marker in the panel **(B)**. Example of NxN matrix use to evaluate unmixing **(C)**. Dot plots of SSC-A vs rare or dim markers in the panel, overlaid with FMO controls to show true positive events **(D)**.

Supplementary Figure 3 Canine samples were batched based on an individual basis **(A)**. After batch correction and clustering, no batch effects were observed based on source of dogs **(B)** or based on condition **(C)**.

Supplementary Figure 4 Feline gating strategy to gate out debris and CD44- cells, aggregates, and dead cells **(A)**. Dot plots of SSC-A vs each marker in the panel **(B)**. Example of NxN matrix use to evaluate unmixing **(C)**. Dot plots of SSC-A vs rare or dim markers in the panel, overlaid with FMO controls to show true positive events **(D)**.

Supplemental Figure 5 Feline samples were batched based on an individual basis **(A**). After batch correction and clustering, no batch effects were observed based on condition **(C)**.

## References

[1] Evans SJM. Flow cytometry in veterinary practice. Vet Clin N Am Small Anim Pract. (2023) 53:89–100. 10.1016/j.cvsm.2022.07.008 36270838

[2] Hengel RL Thaker V Pavlick MV Metcalf JA Dennis G Jr Yang J. Cutting edge: L-selectin (CD62L) expression distinguishes small resting memory CD4+ T cells that preferentially respond to recall antigen. J Immunol. (2003) 170:28–32. 10.4049/jimmunol.170.1.28 12496379

[3] Kawano M Takagi R Tokano M Matsushita S. Adenosine induces IL-31 secretion by T-helper 2 cells: implication for the effect of adenosine on atopic dermatitis and its therapeutic strategy. Biochem Biophys Res Commun. (2023) 645:47–54. 10.1016/j.bbrc.2023.01.038 36680936

[4] McCandless EE Rugg CA Fici GJ Messamore JE Aleo MM Gonzales AJ. Allergen-induced production of IL-31 by canine Th2 cells and identification of immune, skin, and neuronal target cells. Vet Immunol Immunopathol. (2014) 157:42–8. 10.1016/j.vetimm.2013.10.017 24321252

[5] Walsh CM Hill RZ Schwendinger-Schreck J Deguine J Brock EC Kucirek N. Neutrophils promote CXCR3-dependent itch in the development of atopic dermatitis. eLife. (2019) 8:48448. 10.7554/eLife.48448 31631836 PMC6884397

[6] Ko MJ Tsai WC Peng YS Hsu SP Pai MF Yang JY. Altered monocytic phenotypes are associated with uraemic pruritus in patients receiving haemodialysis. Acta Dermatovenereol. (2021) 101:adv00479. 10.2340/00015555-3841 34043016 PMC9380278

[7] Tseng P-Y Hoon MA. Oncostatin M can sensitize sensory neurons in inflammatory pruritus. (2021) 13:eabe3037. 10.1126/scitranslmed.abe3037 34757808 PMC9595590

[8] Suehiro M Numata T Saito R Yanagida N Ishikawa C Uchida K. Oncostatin M suppresses IL31RA expression in dorsal root ganglia and interleukin-31-induced itching. Front Immunol. (2023) 14:1251031. 10.3389/fimmu.2023.1251031 38035099 PMC10687395

[9] Goulart MR Hlavaty SI Chang YM Polton G Stell A Perry J. Phenotypic and transcriptomic characterization of canine myeloid-derived suppressor cells. Sci Rep. (2019) 9:3574. 10.1038/s41598-019-40285-3 30837603 PMC6400936

[10] Maeda S Murakami K Inoue A Yonezawa T Matsuki N. CCR4 blockade depletes regulatory T cells and prolongs survival in a canine model of bladder cancer. Cancer Immunol Res. (2019) 7:1175–87. 10.1158/2326-6066.CIR-18-0751 31160277

[11] Ciftci O Müller LM Jäggle L-M Lehmann C Kneilmann C Stierstorfer B. Cross-reactivity of human monoclonal antibodies with canine peripheral blood mononuclear cells. Vet Immunol Immunopathol. (2023) 259:110578. 10.1016/j.vetimm.2023.110578 36965292

[12] Sandmaier BM Storb R Bennett KL Appelbaum FR Santos EB. Epitope specificity of CD44 for monoclonal antibody–dependent facilitation of marrow engraftment in a canine model. Blood. (1998) 91:3494–502. 10.1182/blood.V91.9.3494.3494_3494_3502 9558410

[13] Out TA Wang S-Z Rudolph K Bice DE. Local T-cell activation after segmental allergen challenge in the lungs of allergic dogs. (2002) 105:499–508. 10.1046/j.1365-2567.2002.01383.x 11985670 PMC1782676

[14] Hartley AN Tarleton RL. Chemokine receptor 7 (CCR7)-expression and IFNγ production define vaccine-specific canine T-cell subsets. Vet Immunol Immunopathol. (2015) 164:127–36. 10.1016/j.vetimm.2015.02.001 25758065 PMC4387071

[15] Mikkelsen SR Long JM Zhang L Galemore ER VandeWoude S Dean GA. Partial regulatory T cell depletion prior to acute feline immunodeficiency virus infection does not alter disease pathogenesis. PLoS ONE. (2011) 6:e17183. 10.1371/journal.pone.0017183 21364928 PMC3045403

[16] Gebhard DH Dow JL Childers TA Alvelo JI Tompkins MB Tompkins WAF. Progressive expansion of an L-selectin—negative CD8 cell with anti—feline immunodeficiency virus (FIV) suppressor function in the circulation of FIV-infected cats. J Infect Dis. (1999) 180:1503–13. 10.1086/315089 10515809

[17] Zwicklbauer K von la Roche D Krentz D Kolberg L Alberer M Zablotski Y. Adapting the SMART tube technology for flow cytometry in feline full blood samples. (2024) 11:1377414. 10.3389/fvets.2024.1377414 38988976 PMC11234156

[18] Kapoor S Sen S Tsang J Yap QJ Park S Cromarty J. Prognostic utility of the flow cytometry and clonality analysis results for feline lymphomas. Vet Sci. (2024) 11:331. 10.3390/vetsci11080331 39195785 PMC11360694

[19] Korsunsky I Millard N Fan J Slowikowski K Zhang F Wei K. Fast, sensitive, and accurate integration of single cell data with Harmony. Nat Methods. (2019) 1289–96. 10.1038/s41592-019-0619-0 31740819 PMC6884693

[20] Zhang Y Parmigiani G Johnson EW. ComBat-seq: batch effect adjustment for RNA-seq count data. NAR Genom Bioinf . (2020) 2:lqaa078. 10.1093/nargab/lqaa078 33015620 PMC7518324

[21] Johnson WE Li C Rabinovic A. Adjusting batch effects in microarray expression data using empirical Bayes methods. Biostatistics. (2006) 8:118–27. 10.1093/biostatistics/kxj037 16632515

[22] Traag VA Waltman L van Eck NJ. From Louvain to Leiden: guaranteeing well-connected communities. Sci Rep. (2019) 9. 10.1038/s41598-019-41695-z 30914743 PMC6435756

[23] Douze M Guzhva A Deng C Johnson J Szilvasy G Mazaré P-E. The Faiss library. arXiv [preprint]. (2024).39072889

[24] Aleo M Zuehlk K Bratschi K King V McDonald E Deines D. Oral oclacitinib maleate and prednisolone demonstrate similar anti-inflammatory effects in a laboratory model of topical challenge in beagles sensitized to Dermatophagoides farinae. (2024).

[25] Martins GC de Oliveira Melo Júnior OA Botoni LS Nogueira MM da Costa Val AP Blanco BS. Clinical-pathological and immunological biomarkers in dogs with atopic dermatitis. Vet Immunol Immunopathol. (2018) 205:58–64. 10.1016/j.vetimm.2018.10.009 30459002

[26] Verde MT Villanueva-Saz S Loste A Marteles D Pereboom D Conde T. Comparison of circulating CD4(+), CD8(+) lymphocytes and cytokine profiles between dogs with atopic dermatitis and healthy dogs. Res Vet Sci. (2022) 145:13–20. 10.1016/j.rvsc.2022.01.018 35134678

[27] Maina E Devriendt B Cox E. Food allergen-specific sublingual immunotherapy modulates peripheral T cell responses of dogs with adverse food reactions. Vet Immunol Immunopathol. (2019) 212:38–42. 10.1016/j.vetimm.2019.05.003 31213250

[28] Davis LS Oppenheimer-Marks N Bednarczyk JL McIntyre BW Lipsky PE. Fibronectin promotes proliferation of naive and memory T cells by signaling through both the VLA-4 and VLA-5 integrin molecules. J Immunol. (1990) 145:785–93. 10.4049/jimmunol.145.3.785 1695644

[29] Jin Z Shen Y Fujimoto S. Role of alpha4 integrin and its ligand VCAM-1 in the specific extravasation of a tumor-specific TH2 clone into tumor tissue that initiates its rejection. Int J Cancer. (2004) 111:558–67. 10.1002/ijc.20288 15239134

[30] Chiu BC Martin BE Stolberg VR Chensue SW. Cutting edge: central memory CD8 T cells in aged mice are virtual memory cells. J Immunol. (2013) 191:5793–6. 10.4049/jimmunol.1302509 24227783 PMC3858473

[31] Hamann D Baars PA Rep MHG Hooibrink B Kerkhof-Garde SR Klein MR. Phenotypic and functional separation of memory and effector human CD8+ T cells. J Exp Med. (1997) 186:1407–18. 10.1084/jem.186.9.1407 9348298 PMC2199103

[32] Pulko V Davies JS Martinez C Lanteri MC Busch MP Diamond MS. Human memory T cells with a naive phenotype accumulate with aging and respond to persistent viruses. Nat Immunol. (2016) 17:966–75. 10.1038/ni.3483 27270402 PMC4955715

[33] Zhang BX Lyu JC Liu HB Feng DQ Zhang DC Bi XJ. Attenuation of peripheral regulatory T-cell suppression of skin-homing CD8?T cells in atopic dermatitis. Yonsei Med J. (2015) 56:196–203. 10.3349/ymj.2015.56.1.196 25510765 PMC4276756

[34] Zhang YY Wang AX Xu L Shen N Zhu J Tu CX. Characteristics of peripheral blood CD4+CD25+ regulatory T cells and related cytokines in severe atopic dermatitis. Eur J Dermatol. (2016) 26:240–6. 10.1684/ejd.2015.2709 27184163

[35] Hauck V Hügli P Meli ML Rostaher A Fischer N Hofmann-Lehmann R. Increased numbers of FoxP3-expressing CD4+ CD25+ regulatory T cells in peripheral blood from dogs with atopic dermatitis and its correlation with disease severity. Vet Dermatol. (2016) 27:26–e9. 10.1111/vde.12279 26748886

[36] Beccati M Martini V Comazzi S Fanton N Cornegliani L. Lymphocyte subpopulations and Treg cells in dogs with atopic dermatitis receiving ciclosporin therapy: a prospective study. Vet Dermatol. (2016) 27:17–e5. 10.1111/vde.12277 26660308

[37] Herrmann I Mamo LB Holmes J Mohammed JP Murphy KM Bizikova P. Long-term effects of ciclosporin and oclacitinib on mediators of tolerance, regulatory T-cells, IL-10 and TGF-β, in dogs with atopic dermatitis. Vet Dermatol. (2023) 34:107–14. 10.1111/vde.13140 36482868

[38] Castillo-González R Cibrian D Sánchez-Madrid F. Dissecting the complexity of γδ T-cell subsets in skin homeostasis, inflammation, and malignancy. J Allergy Clin Immunol. (2021) 147:2030–42. 10.1016/j.jaci.2020.11.023 33259837

[39] Spidale NA Malhotra N Frascoli M Sylvia K Miu B Freeman C. Neonatal-derived IL-17 producing dermal γδ T cells are required to prevent spontaneous atopic dermatitis. eLife. (2020) 9:51188. 10.7554/eLife.51188 32065580 PMC7025821

[40] Sparling BA Moss N Kaur G Clark D Hawkins RD Drechsler Y. Unique cell subpopulations and disease progression markers in canines with atopic dermatitis. J Immunol. (2022) 209:1379–88. 10.4049/jimmunol.2200304 36165204

[41] Tatsuno K Fujiyama T Yamaguchi H Waki M Tokura Y TSLP. Directly interacts with skin-homing Th2 cells highly expressing its receptor to enhance IL-4 production in atopic dermatitis. J Investig Dermatol. (2015) 135:3017–24. 10.1038/jid.2015.318 26288354

[42] Roosje PJ Dean GA Willemse T Rutten VP Thepen T. Interleukin 4-producing CD4+ T cells in the skin of cats with allergic dermatitis. Vet Pathol. (2002) 39:228–33. 10.1354/vp.39-2-228 12009060

[43] Roosje PJ van Kooten PJ Thepen T Bihari IC Rutten VP Koeman JP. Increased numbers of CD4+ and CD8+ T cells in lesional skin of cats with allergic dermatitis. Vet Pathol. (1998) 35:268–73. 10.1177/030098589803500405 9684970

[44] Tedder TF Clement LT Cooper MD. Expression of C3d receptors during human B cell differentiation: immunofluorescence analysis with the HB-5 monoclonal antibody. J Immunol. (1984) 133:678–83. 10.4049/jimmunol.133.2.678 6234356

[45] Reynes M Aubert JP Cohen JH Audouin J Tricottet V Diebold J. Human follicular dendritic cells express CR1, CR2, and CR3 complement receptor antigens. J Immunol. (1985) 135:2687–94. 10.4049/jimmunol.135.4.2687 2411809

[46] Molina E García-Gutiérrez L Junco V Perez-Olivares M de Yébenes VG Blanco R. MYC directly transactivates CR2/CD21, the receptor of the Epstein–Barr virus, enhancing the viral infection of Burkitt lymphoma cells. Oncogene. (2023) 42:3358–70. 10.1038/s41388-023-02846-9 37773203

[47] Chiou SH Chow KC Yang CH Chiang SF Lin CH. Discovery of Epstein-Barr virus (EBV)-encoded RNA signal and EBV nuclear antigen leader protein DNA sequence in pet dogs. J Gen Virol. (2005) 86 (Pt 4):899–905. 10.1099/vir.0.80792-0 15784884

[48] Milman G Smith KC Erles K. Serological detection of Epstein-Barr virus infection in dogs and cats. Vet Microbiol. (2011) 150:15–20. 10.1016/j.vetmic.2010.12.013 21242039

[49] Riondato F Poggi A Miniscalco B Sini F Marconato L Martini V. Flow cytometric features of B- and T-lmphocytes in reactive lymph nodes compared to their neoplastic counterparts in dogs. Vet Sci. (2023) 10:374. 10.3390/vetsci10060374 37368760 PMC10305363

[50] Frémeaux-Bacchi V Fischer E Lecoanet-Henchoz S Mani JC Bonnefoy JY Kazatchkine MD. Soluble CD21 (sCD21) forms biologically active complexes with CD23: sCD21 is present in normal plasma as a complex with trimeric CD23 and inhibits soluble CD23-induced IgE synthesis by B cells. Int Immunol. (1998) 10:1459–66. 10.1093/intimm/10.10.1459 9796912

[51] Wolf AA Yáñez A Barman PK Goodridge HS. The ontogeny of monocyte subsets. Front Immunol. (2019) 10:1642. 10.3389/fimmu.2019.01642 31379841 PMC6650567

[52] Li Z Sun C Wang F Wang X Zhu J Luo L. Molecular mechanisms governing circulating immune cell heterogeneity across different species revealed by single-cell sequencing. Clin Transl Med. (2022) 12:e689. 10.1002/ctm2.689 35092700 PMC8800483

[53] O'Leary CA Sedhom M Reeve-Johnson M Mallyon J Irvine KM. Expression profiling feline peripheral blood monocytes identifies a transcriptional signature associated with type two diabetes mellitus. Vet Immunol Immunopathol. (2017) 186:1–8. 10.1016/j.vetimm.2016.12.011 28413044

[54] Ramarapu R Wulcan JM Chang H Moore PF Vernau W Keller SM. Single cell RNA-sequencing of feline peripheral immune cells with V(D)J repertoire and cross species analysis of T lymphocytes. bioRxiv [preprint]. (2024). 10.1101/2024.05.21.595010 39620216 PMC11604454

[55] Roosje PJ Koeman JP Thepen T Willemse T. Mast cells and eosinophils in feline allergic dermatitis: a qualitative and quantitative analysis. J Comp Pathol. (2004) 131:61–9. 10.1016/j.jcpa.2004.01.005 15144800

[56] Roosje PJ Whitaker-Menezes D Goldschmidt MH Moore PF Willemse T Murphy GF. Feline atopic dermatitis. A model for Langerhans cell participation in disease pathogenesis. Am J Pathol. (1997) 151:927–32. 9327725 PMC1858058

[57] Gibbons N Goulart MR Chang YM Efstathiou K Purcell R Wu Y. Phenotypic heterogeneity of peripheral monocytes in healthy dogs. Vet Immunol Immunopathol. (2017) 190:26–30. 10.1016/j.vetimm.2017.06.007 28778319

[58] Rzepecka A Żmigrodzka M Witkowska-Piłaszewicz O Cywińska A Winnicka A. CD4 and MHCII phenotypic variability of peripheral blood monocytes in dogs. PLoS ONE. (2019) 14:e0219214. 10.1371/journal.pone.0219214 31269060 PMC6608971

[59] Ammons DT Harris RA Hopkins LS Kurihara J Weishaar K Dow S. A single-cell RNA sequencing atlas of circulating leukocytes from healthy and osteosarcoma affected dogs. Front Immunol. (2023) 14:1162700. 10.3389/fimmu.2023.1162700 37275879 PMC10235626

[60] Ricklin Gutzwiller ME Moulin HR Zurbriggen A Roosje P Summerfield A. Comparative analysis of canine monocyte- and bone-marrow-derived dendritic cells. Vet Res. (2010) 41:40. 10.1051/vetres/2010012 20167201 PMC2839791

[61] Moore PF Rossitto PV Danilenko DM Wielenga JJ Raff RF Severns E. Monoclonal antibodies specific for canine CD4 and CD8 define functional T-lymphocyte subsets and high-density expression of CD4 by canine neutrophils. Tissue Antigens. (1992) 40:75–85. 10.1111/j.1399-0039.1992.tb01963.x 1412420

[62] Parys M Bavcar S Mellanby RJ Argyle D Kitamura T. Use of multi-color flow cytometry for canine immune cell characterization in cancer. PLoS ONE. (2023) 18:e0279057. 10.1371/journal.pone.0279057 36996049 PMC10062640

[63] Groh V Tani M Harrer A Wolff K Stingl G. Leu-3/T4 expression on epidermal Langerhans cells in normal and diseased skin. J Invest Dermatol. (1986) 86:115–20. 10.1111/1523-1747.ep12284090 3091703

[64] Wood GS Warner NL Warnke RA. Anti-Leu-3/T4 antibodies react with cells of monocyte/macrophage and Langerhans lineage. J Immunol. (1983) 131:212–6. 10.4049/jimmunol.131.1.212 6408171

[65] Jefferies WA Green JR Williams AF. Authentic T helper CD4 (W3/25) antigen on rat peritoneal macrophages. J Exp Med. (1985) 162:117–27. 10.1084/jem.162.1.117 3159821 PMC2187688

[66] Zhen A Krutzik SR Levin BR Kasparian S Zack JA Kitchen SG. CD4 ligation on human blood monocytes triggers macrophage differentiation and enhances HIV infection. J Virol. (2014) 88:9934–46. 10.1128/JVI.00616-14 24942581 PMC4136363

[67] Goulart MR Pluhar GE Ohlfest JR. Identification of myeloid derived suppressor cells in dogs with naturally occurring cancer. PLoS ONE. (2012) 7:e33274. 10.1371/journal.pone.0033274 22428007 PMC3302813

[68] Yokota S Yonezawa T Momoi Y Maeda S. Myeloid derived suppressor cells in peripheral blood can be a prognostic factor in canine transitional cell carcinoma. Vet Immunol Immunopathol. (2024) 269:110716. 10.1016/j.vetimm.2024.110716 38308864

[69] Cao LY Chung JS Teshima T Feigenbaum L Cruz PD Jr Jacobe HT. Myeloid-derived suppressor cells in psoriasis are an expanded population exhibiting diverse T-cell-suppressor mechanisms. J Investig Dermatol. (2016) 136:1801–10. 10.1016/j.jid.2016.02.816 27236103 PMC4992618

[70] Ozbay Kurt FG Lasser S Arkhypov I Utikal J Umansky V. Enhancing immunotherapy response in melanoma: myeloid-derived suppressor cells as a therapeutic target. J Clin Investig. (2023) 133:762. 10.1172/JCI170762 37395271 PMC10313369

