# Supplementary figures and images for: High-parameter immunophenotyping reveals distinct immune cell profiles in pruritic dogs and cats
Source: Front Vet Sci. 2025 Jan 22;11:1498964. doi: 10.3389/fvets.2024.1498964 (PMC11795398; doi:10.3389/fvets.2024.1498964)

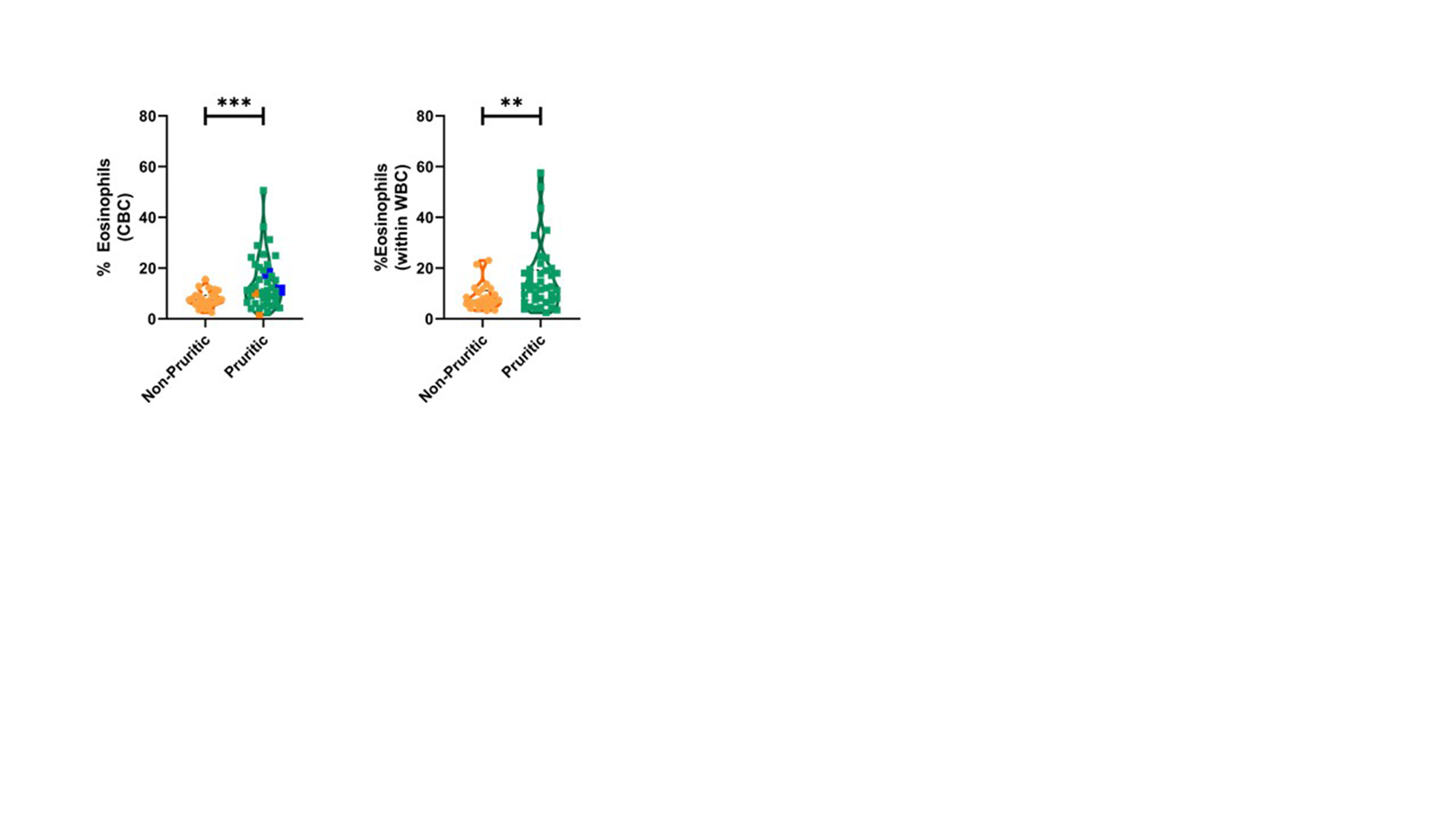

Supplement: Supplementalry Figure 1 — scFlow corroborates CBC data for eosinophils. Violin plots were used to visualize the distribution of data (n = 25 non-pruritic cats (orange); n = 42 pruritic cats (green); within the pruritic cats, cats that had food allergy are colored dark orange and cats with known fleas are colored in blue). Statistical analysis was performed by unpaired t test with Welch's correction. Two-tailed p-values are reported: ***p = 0.0005; **p = 0.0019. [file Image_1.JPEG]

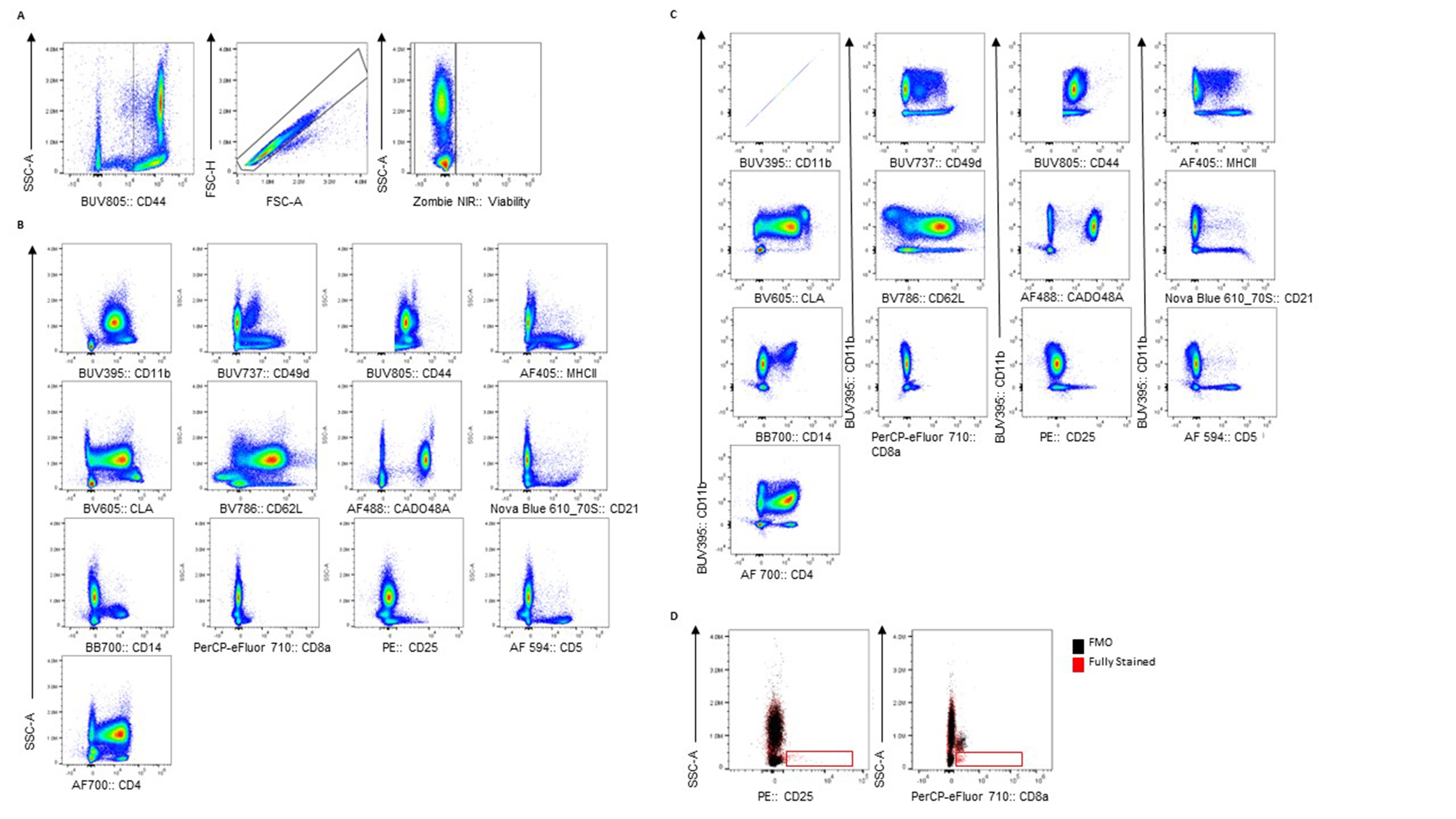

Supplement: Supplementary Figure 2 — Canine gating strategy to gate out debris and CD44- cells, aggregates, and dead cells (A). Dot plots of SSC-A vs each marker in the panel (B). Example of NxN matrix use to evaluate unmixing (C). Dot plots of SSC-A vs rare or dim markers in the panel, overlaid with FMO controls to show true positive events (D). [file Image_2.JPEG]

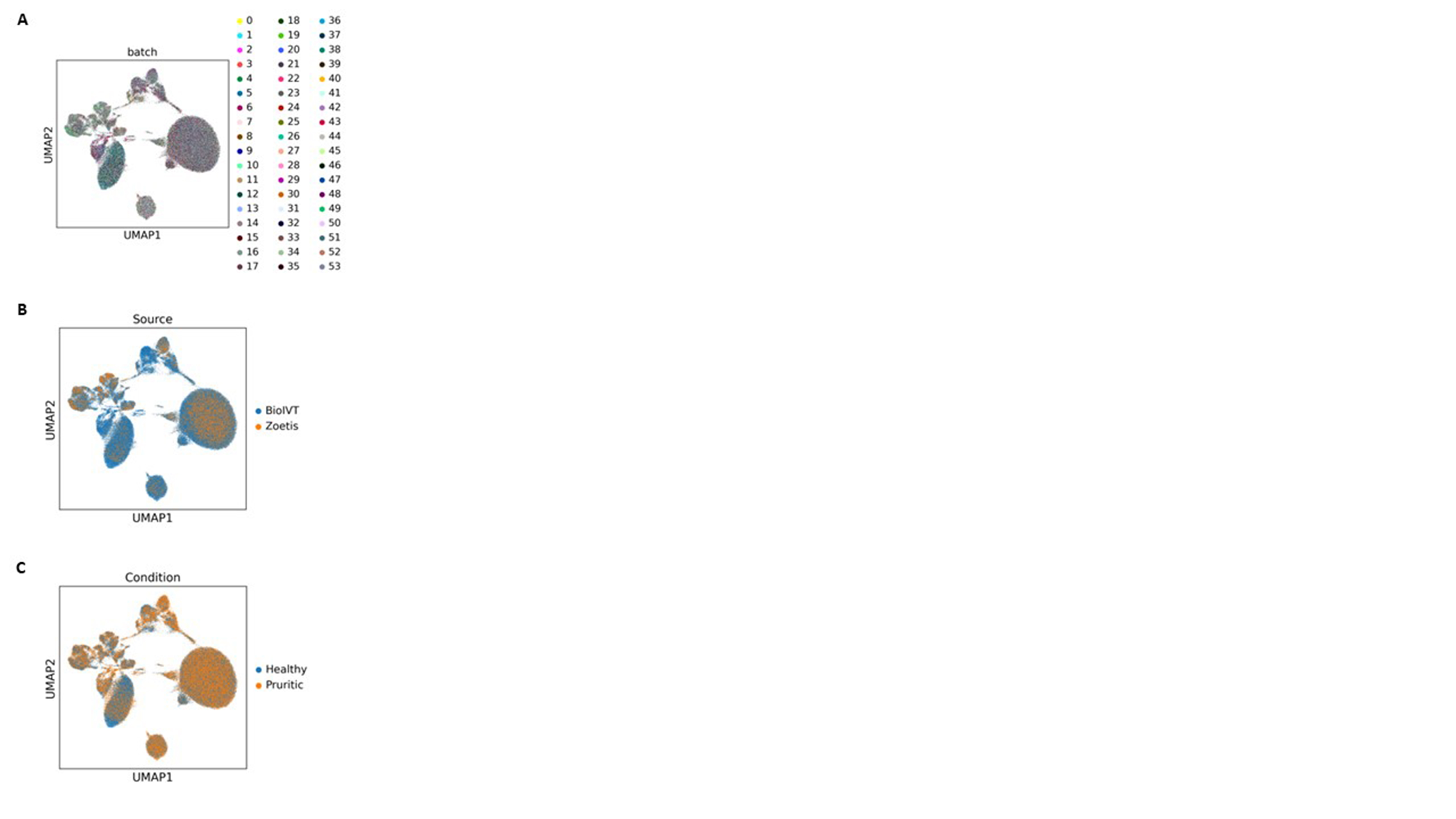

Supplement: Supplementary Figure 3 — Canine samples were batched based on an individual basis (A). After batch correction and clustering, no batch effects were observed based on source of dogs (B) or based on condition (C). [file Image_3.JPEG]

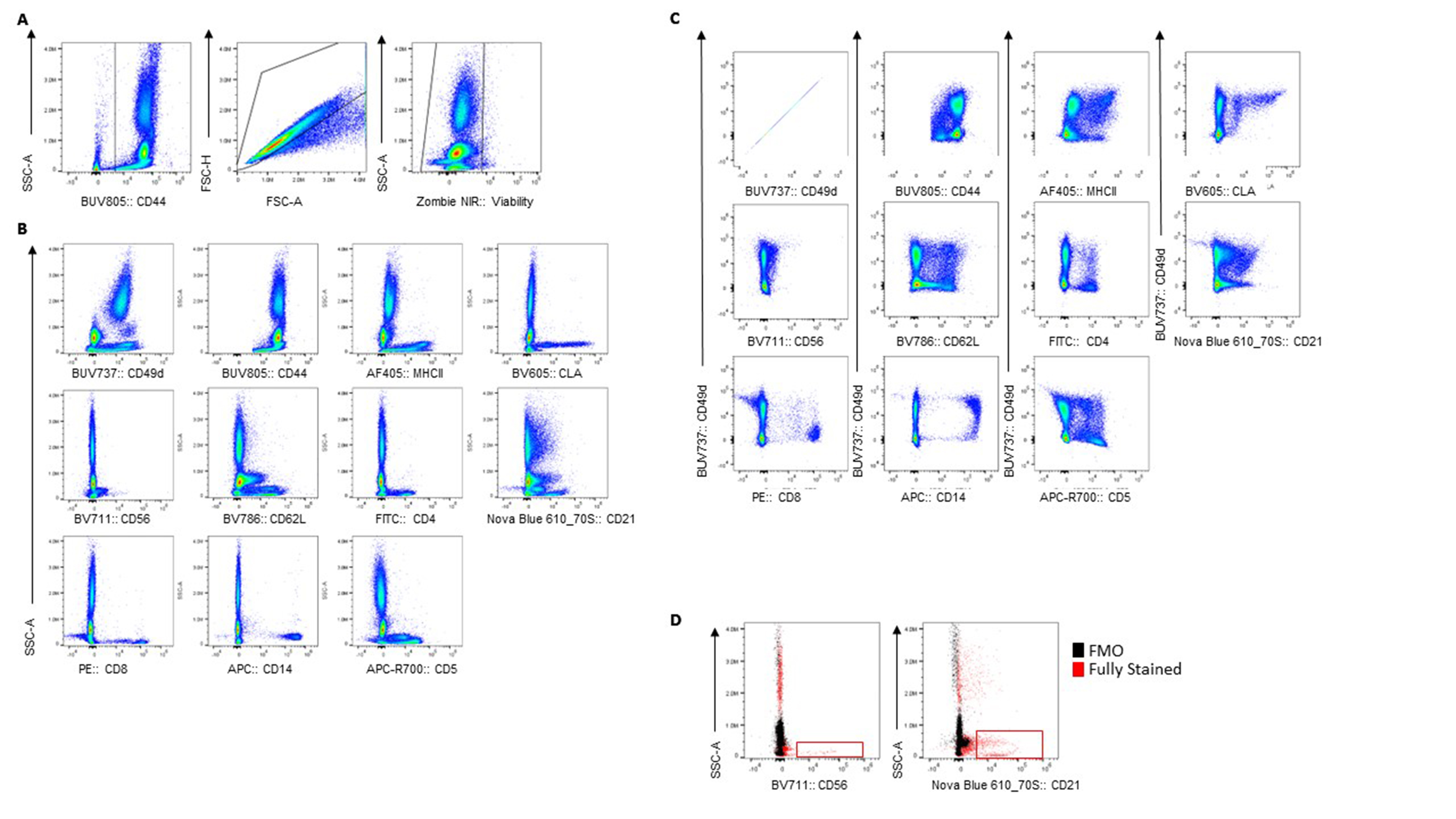

Supplement: Supplementary Figure 4 — Feline gating strategy to gate out debris and CD44- cells, aggregates, and dead cells (A). Dot plots of SSC-A vs each marker in the panel (B). Example of NxN matrix use to evaluate unmixing (C). Dot plots of SSC-A vs rare or dim markers in the panel, overlaid with FMO controls to show true positive events (D). [file Image_4.JPEG]

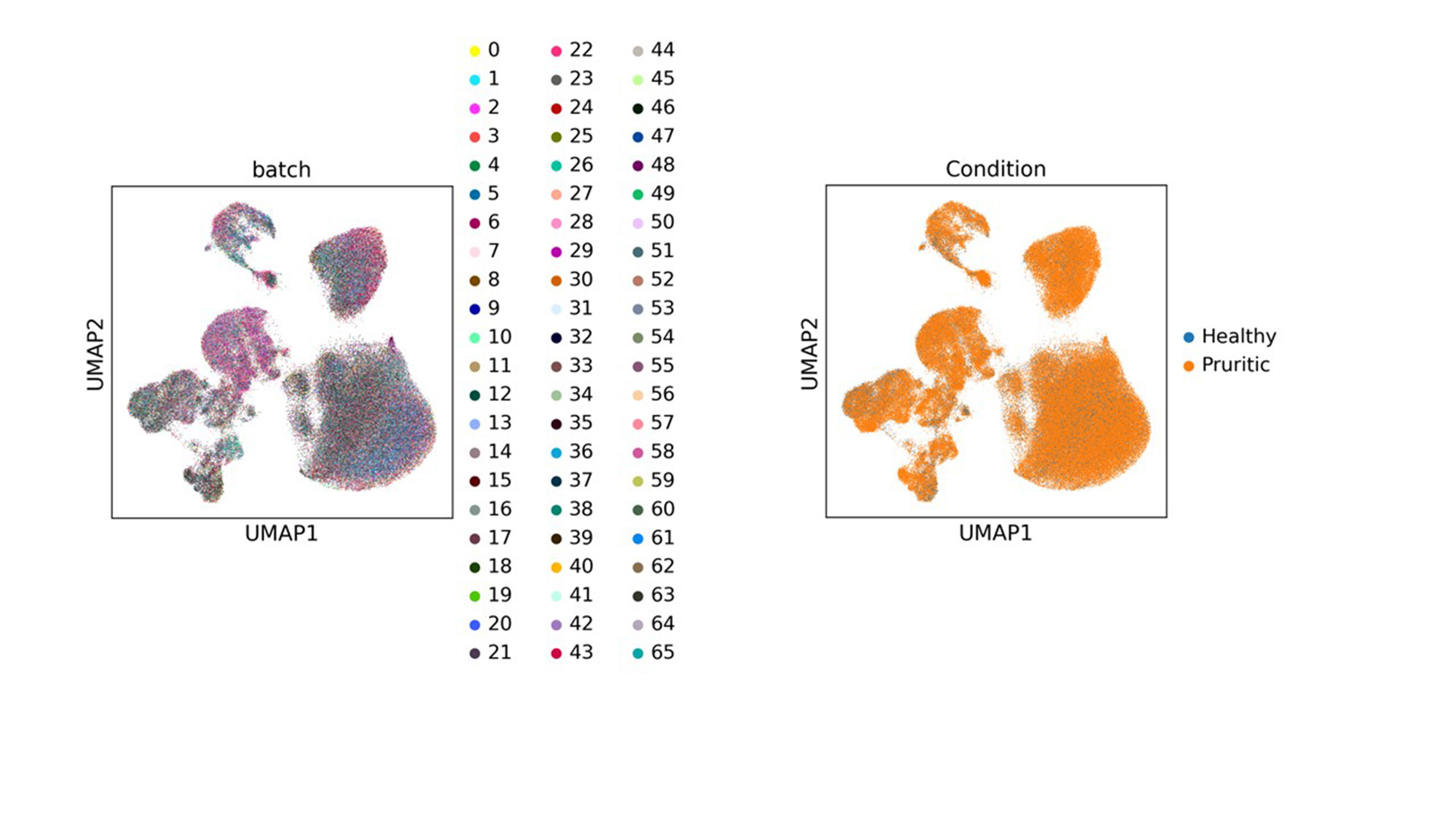

Supplement: Supplemental Figure 5 — Feline samples were batched based on an individual basis (A). After batch correction and clustering, no batch effects were observed based on condition (C). [file Image_5.JPEG]
