# Supplementary material for: High-parameter immunophenotyping reveals distinct immune cell profiles in pruritic dogs and cats
Source: Front Vet Sci. 2025 Jan 22;11:1498964. doi: 10.3389/fvets.2024.1498964 (PMC11795398; doi:10.3389/fvets.2024.1498964)
Supplement: Supplementary file 6 [file Table_1.docx]

## Supplemental Material – Python Packages

| PyPI Package | Version | Description |
| --- | --- | --- |
| aiohttp | 3.8.5 | Async http client/server framework (asyncio) |
| aiosignal | 1.3.1 | aiosignal: a list of registered asynchronous callbacks |
| anndata | 0.9.2 | Annotated data. |
| annoy | 1.17.3 | Approximate Nearest Neighbors in C++/Python optimized for memory usage and loading/saving to disk. |
| anyio | 4.0.0 | High level compatibility layer for multiple asynchronous event loop implementations |
| anytree | 2.9.0 | Powerful and Lightweight Python Tree Data Structure with various plugins |
| argon2-cffi | 23.1.0 | Argon2 for Python |
| argon2-cffi-bindings | 21.2.0 | Low-level CFFI bindings for Argon2 |
| arrow | 1.2.3 | Better dates & times for Python |
| asttokens | 2.4.0 | Annotate AST trees with source code positions |
| async-timeout | 4.0.3 | Timeout context manager for asyncio programs |
| attrs | 23.1.0 | Classes Without Boilerplate |
| backcall | 0.2.0 | Specifications for callback functions passed in to an API |
| bbknn | 1.6.0 | Batch balanced KNN |
| beautifulsoup4 | 4.12.2 | Screen-scraping library |
| black | 23.9.1 | The uncompromising code formatter. |
| bleach | 6.0.0 | An easy safelist-based HTML-sanitizing tool. |
| bokeh | 2.4.3 | Interactive plots and applications in the browser from Python |
| certifi | 2023.7.22 | Python package for providing Mozilla's CA Bundle. |
| cffi | 1.15.1 | Foreign Function Interface for Python calling C code. |
| charset-normalizer | 3.2.0 | The Real First Universal Charset Detector. Open, modern and actively maintained alternative to Chardet. |
| click | 8.1.7 | Composable command line interface toolkit |
| cloudpickle | 2.2.1 | Extended pickling support for Python objects |
| ClusterEnsembles | 0.2.7 | ClusterEnsembles A Python package for cluster ensembles |
| combat | 0.3.3 | pyComBat, a Python tool for batch effects correction in high-throughput molecular data using empirical Bayes... |
| comm | 0.1.4 | Jupyter Python Comm implementation, for usage in ipykernel, xeus-python etc. |
| contourpy | 1.1.0 | Python library for calculating contours of 2D quadrilateral grids |
| cycler | 0.11.0 | Composable style cycles |
| cython | 3.0.2 | The Cython compiler for writing C extensions in the Python language. |
| dask | 2023.9.1 | Parallel PyData with Task Scheduling |
| dask-jobqueue | 0.8.2 | Deploy Dask on job queuing systems like PBS, Slurm, SGE or LSF |
| debugpy | 1.8.0 | An implementation of the Debug Adapter Protocol for Python |
| decorator | 5.1.1 | Decorators for Humans |
| defusedxml | 0.7.1 | XML bomb protection for Python stdlib modules |
| distributed | 2023.9.1 | Distributed scheduler for Dask |
| dnspython | 2.5.0 | DNS toolkit |
| et-xmlfile | 1.1.0 | An implementation of lxml.xmlfile for the standard library |
| exceptiongroup | 1.1.3 | Backport of PEP 654 (exception groups) |
| executing | 1.2.0 | Get the currently executing AST node of a frame, and other information |
| faiss-cpu | 1.7.4 | A library for efficient similarity search and clustering of dense vectors. |
| fastapi | 0.95.2 | FastAPI framework, high performance, easy to learn, fast to code, ready for production |
| fastjsonschema | 2.18.0 | Fastest Python implementation of JSON schema |
| filelock | 3.12.4 | A platform independent file lock. |
| FlowGrid | v1.1.2 | FlowGrid |
| flowio | 1.1.1 | FlowIO is a Python library for reading / writing Flow Cytometry Standard (FCS) files |
| flowkit | 1.0.1 | Flow Cytometry Toolkit |
| flowutils | 1.0.0 | Flow Cytometry Standard Utilities |
| fonttools | 4.42.1 | Tools to manipulate font files |
| fqdn | 1.5.1 | Validates fully-qualified domain names against RFC 1123, so that they are acceptable to modern bowsers |
| frozenlist | 1.4.0 | A list-like structure which implements collections.abc.MutableSequence |
| fsspec | 2023.9.0 | File-system specification |
| h11 | 0.14.0 | A pure-Python, bring-your-own-I/O implementation of HTTP/1.1 |
| h5py | 3.9.0 | Read and write HDF5 files from Python |
| harmonypy | 0.0.9 | A data integration algorithm. |
| httpcore | 0.18.0 | A minimal low-level HTTP client. |
| httpx | 0.25.0 | The next generation HTTP client. |
| idna | 3.4 | Internationalized Domain Names in Applications (IDNA) |
| igraph | 0.10.8 | High performance graph data structures and algorithms |
| importlib-metadata | 6.8.0 | Read metadata from Python packages |
| ipykernel | 6.25.2 | IPython Kernel for Jupyter |
| ipython | 8.15.0 | IPython: Productive Interactive Computing |
| isoduration | 20.11.0 | Operations with ISO 8601 durations |
| jedi | 0.19.0 | An autocompletion tool for Python that can be used for text editors. |
| jinja2 | 3.1.2 | A very fast and expressive template engine. |
| joblib | 1.3.2 | Lightweight pipelining with Python functions |
| jsonpointer | 2.4 | Identify specific nodes in a JSON document (RFC 6901) |
| jsonschema | 4.19.0 | An implementation of JSON Schema validation for Python |
| jsonschema-specifications | 2023.7.1 | The JSON Schema meta-schemas and vocabularies, exposed as a Registry |
| jupyter-client | 8.3.1 | Jupyter protocol implementation and client libraries |
| jupyter-core | 5.3.1 | Jupyter core package. A base package on which Jupyter projects rely. |
| jupyter-events | 0.7.0 | Jupyter Event System library |
| jupyter-server | 2.7.3 | The backendâ€”i.e. core services, APIs, and REST endpointsâ€”to Jupyter web applications. |
| jupyter-server-proxy | 4.0.0 | A JupyterLab extension accompanying the PyPI package jupyter-server-proxy adding launcher items for configur... |
| jupyter-server-terminals | 0.4.4 | A Jupyter Server Extension Providing Terminals. |
| jupyterlab-pygments | 0.2.2 | Pygments theme using JupyterLab CSS variables |
| kahypar | 1.3.5 | Python Inferface for the Karlsruhe Hypergraph Partitioning Framework (KaHyPar) |
| kiwisolver | 1.4.5 | A fast implementation of the Cassowary constraint solver |
| leidenalg | 0.10.1 | Leiden is a general algorithm for methods of community detection in large networks. |
| llvmlite | 0.40.1 | lightweight wrapper around basic LLVM functionality |
| locket | 1.0.0 | File-based locks for Python on Linux and Windows |
| loky | 3.4.1 | A robust implementation of concurrent.futures.ProcessPoolExecutor |
| lxml | 4.9.3 | Powerful and Pythonic XML processing library combining libxml2/libxslt with the ElementTree API. |
| markdown-it-py | 3.0.0 | Python port of markdown-it. Markdown parsing, done right! |
| markupsafe | 2.1.3 | Safely add untrusted strings to HTML/XML markup. |
| matplotlib | 3.7.3 | Python plotting package |
| matplotlib-inline | 0.1.6 | Inline Matplotlib backend for Jupyter |
| mdurl | 0.1.2 | Markdown URL utilities |
| mistune | 3.0.1 | A sane and fast Markdown parser with useful plugins and renderers |
| mpmath | 1.3.0 | Python library for arbitrary-precision floating-point arithmetic |
| msgpack | 1.0.5 | MessagePack serializer |
| multidict | 6.0.4 | multidict implementation |
| mypy | 1.5.1 | Optional static typing for Python |
| mypy-extensions | 1.0.0 | Type system extensions for programs checked with the mypy type checker. |
| natsort | 8.4.0 | Simple yet flexible natural sorting in Python. |
| nbclient | 0.8.0 | A client library for executing notebooks. Formerly nbconvert's ExecutePreprocessor. |
| nbconvert | 7.8.0 | Converting Jupyter Notebooks |
| nbformat | 5.9.2 | The Jupyter Notebook format |
| nest-asyncio | 1.5.7 | Patch asyncio to allow nested event loops |
| networkx | 3.1 | Python package for creating and manipulating graphs and networks |
| nodeenv | 1.8.0 | Node.js virtual environment builder |
| numba | 0.57.1 | compiling Python code using LLVM |
| numpy | 1.24.4 | Fundamental package for array computing in Python |
| openpyxl | 3.1.2 | A Python library to read/write Excel 2010 xlsx/xlsm files |
| overrides | 7.4.0 | A decorator to automatically detect mismatch when overriding a method. |
| packaging | 23.1 | Core utilities for Python packages |
| pandas | 1.5.3 | Powerful data structures for data analysis, time series, and statistics |
| pandocfilters | 1.5.0 | Utilities for writing pandoc filters in python |
| parso | 0.8.3 | A Python Parser |
| partd | 1.4.0 | Appendable key-value storage |
| pathspec | 0.11.2 | Utility library for gitignore style pattern matching of file paths. |
| patsy | 0.5.3 | A Python package for describing statistical models and for building design matrices. |
| pexpect | 4.8.0 | Pexpect allows easy control of interactive console applications. |
| phenograph | 1.5.7 | Graph-based clustering for high-dimensional single-cell data |
| pickleshare | 0.7.5 | Tiny 'shelve'-like database with concurrency support |
| pillow | 10.0.0 | Python Imaging Library (Fork) |
| platformdirs | 3.10.0 | A small Python package for determining appropriate platform-specific dirs, e.g. a 'user data dir'. |
| prisma | 0.8.2 | Prisma Client Python is an auto-generated and fully type-safe database client |
| prometheus-client | 0.17.1 | Python client for the Prometheus monitoring system. |
| prompt-toolkit | 3.0.39 | Library for building powerful interactive command lines in Python |
| protobuf | 4.24. | 3 |
| psutil | 5.9.5 | Cross-platform lib for process and system monitoring in Python. |
| ptyprocess | 0.7.0 | Run a subprocess in a pseudo terminal |
| pure-eval | 0.2.2 | Safely evaluate AST nodes without side effects |
| pycparser | 2.21 | C parser in Python |
| pydantic | 1.10.12 | Data validation and settings management using python type hints |
| pygments | 2.16.1 | Pygments is a syntax highlighting package written in Python. |
| pyhumps | 3.8.0 | ðŸ« Convert strings (and dictionary keys) between snake case, camel case and pascal case in Python. Inspired ... |
| pymetis | 2023.1.1 | A Graph Partitioning Package |
| pymongo | 4.6.1 | Python driver for MongoDB <http://www.mongodb.org> |
| pynndescent | 0.5.10 | Nearest Neighbor Descent |
| pyparsing | 3.1.1 | pyparsing module - Classes and methods to define and execute parsing grammars |
| python-dateutil | 2.8.2 | Extensions to the standard Python datetime module |
| python-dotenv | 1.0.0 | Read key-value pairs from a .env file and set them as environment variables |
| python-json-logger | 2.0.7 | A python library adding a json log formatter |
| pytz | 2023.3.post1 | World timezone definitions, modern and historical |
| pyyaml | 6.0.1 | YAML parser and emitter for Python |
| pyzmq | 25.1.1 | Python bindings for 0MQ |
| ray | 2.7.0 | Ray provides a simple, universal API for building distributed applications. |
| referencing | 0.30.2 | JSON Referencing + Python |
| requests | 2.31.0 | Python HTTP for Humans. |
| rfc3339-validator | 0.1.4 | A pure python RFC3339 validator |
| rfc3986-validator | 0.1.1 | Pure python rfc3986 validator |
| rich | 13.5.2 | Render rich text, tables, progress bars, syntax highlighting, markdown and more to the terminal |
| rpds-py | 0.10.3 | Python bindings to Rust's persistent data structures (rpds) |
| scanpy | 1.9.5 | Single-Cell Analysis in Python. |
| scikit-learn | 1.3.0 | A set of python modules for machine learning and data mining |
| scikit-misc | 0.2.0 | Miscellaneous tools for scientific computing. |
| scipy | 1.9.3 | Fundamental algorithms for scientific computing in Python |
| seaborn | 0.11.2 | seaborn: statistical data visualization |
| send2trash | 1.8.2 | Send file to trash natively under Mac OS X, Windows and Linux |
| session-info | 1.0.0 | session_info outputs version information for modules loaded in the current session, Python, and the OS. |
| setuptools | 68.2.2 | Easily download, build, install, upgrade, and uninstall Python packages |
| setuptools-scm | 7.1.0 | the blessed package to manage your versions by scm tags |
| simpervisor | 1.0.0 | Simple async process supervisor |
| six | 1.16.0 | Python 2 and 3 compatibility utilities |
| sniffio | 1.3.0 | Sniff out which async library your code is running under |
| sortedcontainers | 2.4.0 | Sorted Containers -- Sorted List, Sorted Dict, Sorted Set |
| soupsieve | 2.5 | A modern CSS selector implementation for Beautiful Soup. |
| stack-data | 0.6.2 | Extract data from python stack frames and tracebacks for informative displays |
| starlette | 0.27.0 | The little ASGI library that shines. |
| statsmodels | 0.14.0 | Statistical computations and models for Python |
| stdlib-list | 0.9.0 | A list of Python Standard Libraries (2.7 through 3.9). |
| sympy | 1.12 | Computer algebra system (CAS) in Python |
| tblib | 2.0.0 | Traceback serialization library. |
| terminado | 0.17.1 | Tornado websocket backend for the Xterm.js Javascript terminal emulator library. |
| texttable | 1.6.7 | module to create simple ASCII tables |
| threadpoolctl | 3.2.0 | threadpoolctl |
| tinycss2 | 1.2.1 | A tiny CSS parser |
| tomli | 2.0.1 | A lil' TOML parser |
| tomlkit | 0.12.1 | Style preserving TOML library |
| toolz | 0.12.0 | List processing tools and functional utilities |
| torch | 2.0.1+cpu | Tensors and Dynamic neural networks in Python with strong GPU acceleration |
| torchaudio | 2.0.2+cpu | An audio package for PyTorch |
| torchvision | 0.15.2+cpu | image and video datasets and models for torch deep learning |
| tornado | 6.3.3 | Tornado is a Python web framework and asynchronous networking library, originally developed at FriendFeed. |
| tqdm | 4.66.1 | Fast, Extensible Progress Meter |
| traitlets | 5.10.0 | Traitlets Python configuration system |
| typing | 3.7.4.3 | Type Hints for Python |
| typing-extensions | 4.7.1 | Backported and Experimental Type Hints for Python 3.7+ |
| umap-learn | 0.5.3 | Uniform Manifold Approximation and Projection |
| uri-template | 1.3.0 | RFC 6570 URI Template Processor |
| urllib3 | 2.0.4 | HTTP library with thread-safe connection pooling, file post, and more. |
| uvicorn | 0.21.1 | The lightning-fast ASGI server. |
| wcwidth | 0.2.6 | Measures the displayed width of unicode strings in a terminal |
| webcolors | 1.13 | A library for working with the color formats defined by HTML and CSS. |
| webencodings | 0.5.1 | Character encoding aliases for legacy web content |
| websocket-client | 1.6.3 | WebSocket client for Python with low level API options |
| yarl | 1.9.2 | Yet another URL library |
| zict | 3.0.0 | Mutable mapping tools |
| zipp | 3.16.2 | Backport of pathlib-compatible object wrapper for zip files |
